# Inhibited Methanogenesis in the Rumen of Cattle: Microbial Metabolism in Response to Supplemental 3-Nitrooxypropanol and Nitrate

**DOI:** 10.3389/fmicb.2021.705613

**Published:** 2021-07-27

**Authors:** Henk J. van Lingen, James G. Fadel, David R. Yáñez-Ruiz, Maik Kindermann, Ermias Kebreab

**Affiliations:** ^1^Department of Animal Science, University of California, Davis, Davis, CA, United States; ^2^Estación Experimental del Zaidín (CSIC), Granada, Spain; ^3^Research and Development, DSM Nutritional Products, Basel, Switzerland

**Keywords:** 3-NOP, nitrite, cattle, feed supplement, bacteria, archaea, methane

## Abstract

3-Nitrooxypropanol (3-NOP) supplementation to cattle diets mitigates enteric CH_4_ emissions and may also be economically beneficial at farm level. However, the wider rumen metabolic response to methanogenic inhibition by 3-NOP and the $\text{N}{\text{O}}_{2}^{-}$ intermediary metabolite requires further exploration. Furthermore, $\text{N}{\text{O}}_{3}^{-}$ supplementation potently decreases CH_4_ emissions from cattle. The reduction of $\text{N}{\text{O}}_{3}^{-}$ utilizes H_2_ and yields $\text{N}{\text{O}}_{2}^{-}$, the latter of which may also inhibit rumen methanogens, although a different mode of action than for 3-NOP and its $\text{N}{\text{O}}_{2}^{-}$ derivative was hypothesized. Our objective was to explore potential responses of the fermentative and methanogenic metabolism in the rumen to 3-NOP, $\text{N}{\text{O}}_{3}^{-}$ and their metabolic derivatives using a dynamic mechanistic modeling approach. An extant mechanistic rumen fermentation model with state variables for carbohydrate substrates, bacteria and protozoa, gaseous and dissolved fermentation end products and methanogens was extended with a state variable of either 3-NOP or $\text{N}{\text{O}}_{3}^{-}$. Both new models were further extended with a $\text{N}{\text{O}}_{2}^{-}$ state variable, with $\text{N}{\text{O}}_{2}^{-}$ exerting methanogenic inhibition, although the modes of action of 3-NOP-derived and $\text{N}{\text{O}}_{3}^{-}$-derived $\text{N}{\text{O}}_{2}^{-}$ are different. Feed composition and intake rate (twice daily feeding regime), and supplement inclusion were used as model inputs. Model parameters were estimated to experimental data collected from the literature. The extended 3-NOP and $\text{N}{\text{O}}_{3}^{-}$ models both predicted a marked peak in H_2_ emission shortly after feeding, the magnitude of which increased with higher doses of supplement inclusion. The H_2_ emission rate appeared positively related to decreased acetate proportions and increased propionate and butyrate proportions. A decreased CH_4_ emission rate was associated with 3-NOP and $\text{N}{\text{O}}_{3}^{-}$ supplementation. Omission of the $\text{N}{\text{O}}_{2}^{-}$ state variable from the 3-NOP model did not change the overall dynamics of H_2_ and CH_4_ emission and other metabolites. However, omitting the $\text{N}{\text{O}}_{2}^{-}$ state variable from the $\text{N}{\text{O}}_{3}^{-}$ model did substantially change the dynamics of H_2_ and CH_4_ emissions indicated by a decrease in both H_2_ and CH_4_ emission after feeding. Simulations do not point to a strong relationship between methanogenic inhibition and the rate of $\text{N}{\text{O}}_{3}^{-}$ and $\text{N}{\text{O}}_{2}^{-}$ formation upon 3-NOP supplementation, whereas the metabolic response to $\text{N}{\text{O}}_{3}^{-}$ supplementation may largely depend on methanogenic inhibition by $\text{N}{\text{O}}_{2}^{-}$.

## 1. Introduction

Animal agriculture emits about 7.1 gigatonnes of CO_2_ equivalents of greenhouse gases per year, which represents approximately 14.5% of total global anthropogenic greenhouse gas emissions in 2005 (Gerber et al., 2013). Dairy and beef cattle emitted 4.6 gigatonnes CO_2_ equivalents, of which CH_4_ from enteric fermentation contributed about 45%. To decrease the latter enteric source of greenhouse gas emission, various dietary supplements with a potential inhibiting effect on ruminal methanogenesis have been tested. 3-nitrooxypropanol (3-NOP) is one of the most effective dietary supplements that was tested for cattle (e.g., Hristov et al., 2015), and may also be economically beneficial (Alvarez-Hess et al., 2019). The mode of action of 3-NOP was elucidated to be the inhibition of methyl co-enzyme-M reductase (MCR), with clear indications that $\text{N}{\text{O}}_{2}^{-}$ can be metabolized from 3-NOP and inhibit methanogenesis by blocking MCR activity as well (Duin et al., 2016). However, the wider effects of 3-NOP and $\text{N}{\text{O}}_{2}^{-}$ on methanogenic archaea in the rumen and the implications for the dynamics of ruminal metabolites require a more thorough exploration.

Nitrate is another dietary supplement (commonly in the form of a calcium salt, sometimes a sodium or potassium salt) that has been observed to decrease enteric CH_4_ from cattle substantially and persistently (Van Zijderveld et al., 2011), although there seem no on-farm economical benefits (Alvarez-Hess et al., 2019). Nitrate is primarily reduced to NH_3_ by ruminal bacteria, which may result in the utilization of four equivalents of H_2_ per equivalent of $\text{N}{\text{O}}_{3}^{-}$. This reduction reaction causes less H_2_ available for CH_4_ production by the methanogens. However, $\text{N}{\text{O}}_{3}^{-}$ supplementation to dairy cattle diets was reported to increase H_2_ emissions (Olijhoek et al., 2016). The latter increase was explained by $\text{N}{\text{O}}_{3}^{-}$ being reduced to $\text{N}{\text{O}}_{2}^{-}$, with $\text{N}{\text{O}}_{2}^{-}$ inhibiting the methanogenic metabolism (Latham et al., 2016). Therefore, the presence of $\text{N}{\text{O}}_{2}^{-}$ as an intermediate in the reduction of $\text{N}{\text{O}}_{3}^{-}$ to NH_3_ may contribute to the CH_4_ suppressing effect of $\text{N}{\text{O}}_{3}^{-}$ supplementation to cattle diets as well.

Various ruminal bacteria possess and express genes that result in the employment of periplasmic $\text{N}{\text{O}}_{3}^{-}$ and $\text{N}{\text{O}}_{2}^{-}$ reductases (Kern and Simon, 2009; Yang et al., 2016). The methanogens that reside in the rumen, however, were not observed to transcribe genes that encode for $\text{N}{\text{O}}_{3}^{-}$ and $\text{N}{\text{O}}_{2}^{-}$ reductases (Greening et al., 2019). Lack of these reductases may suggest that the conversion of 3-NOP into $\text{N}{\text{O}}_{2}^{-}$ inside methanogenic cells proceeds spontaneously or is catalyzed by different enzymes, which aligns with the formation of $\text{N}{\text{O}}_{3}^{-}$ and $\text{N}{\text{O}}_{2}^{-}$ upon the inactivation of the MCR enzyme (Duin et al., 2016). Although 3-NOP is transported across the methanogenic cell membrane, no evidence for $\text{N}{\text{O}}_{2}^{-}$ transportation across the methanogenic cell membrane is known to the authors. If $\text{N}{\text{O}}_{2}^{-}$ is transported across the methanogenic cell membrane, the $\text{N}{\text{O}}_{2}^{-}$ derived from $\text{N}{\text{O}}_{3}^{-}$ may even inhibit CH_4_ production completely by blocking MCR at the commonly used dietary inclusion rates of $\text{N}{\text{O}}_{3}^{-}$, which is not commonly observed. On a molar basis, the relatively low inclusion rates of 3-NOP compared to $\text{N}{\text{O}}_{3}^{-}$ will likely result in lower $\text{N}{\text{O}}_{2}^{-}$ production. Therefore, the mechanisms by which $\text{N}{\text{O}}_{2}^{-}$ derived from $\text{N}{\text{O}}_{3}^{-}$ and 3-NOP act on archaea appear different, with 3-NOP derived $\text{N}{\text{O}}_{2}^{-}$ exerting its methanogenic inhibition inside the cell and $\text{N}{\text{O}}_{3}^{-}$ derived $\text{N}{\text{O}}_{2}^{-}$ potentially exerting methanogenic inhibition outside the cell.

Besides metabolic conversions and their enzyme kinetic implications, several studies suggested the inhibiting effect of 3-NOP and $\text{N}{\text{O}}_{3}^{-}$ on ruminal methanogenesis to be partly thermodynamically controlled (Van Zijderveld et al., 2011; Dijkstra et al., 2018). Both 3-NOP and $\text{N}{\text{O}}_{3}^{-}$ were found to increase H_2_ emission, suggesting thermodynamic inhibition of NADH oxidation in fermentative microbes in the rumen (Van Lingen et al., 2016). This thermodynamic inhibition results in a shift from acetate to more propionate production, which decreases the yield of H_2_ and next the yield of CH_4_. The objective of this study is to explore putative mechanisms of methanogenic inhibition by 3-NOP and $\text{N}{\text{O}}_{3}^{-}$ and their implications for the dynamics of microbial fermentation in the bovine rumen using dynamic mechanistic modeling approaches. For this objective, an existing dynamic mechanistic model of microbial substrate degradation that incorporated various metabolic pathways (Van Lingen et al., 2019) is extended with putative kinetic downregulation mechanisms of methanogenesis by 3-NOP, $\text{N}{\text{O}}_{3}^{-}$ and their derivatives. These newly developed modeling approaches also enable the evaluation of the thermodynamic control of H_2_ partial pressure (*p*_H_2__) on volatile fatty acid (VFA) fermentation pathways via the NAD^+^ to NADH ratio in fermentative microbes upon the supplementation of feed with 3-NOP and $\text{N}{\text{O}}_{3}^{-}$.

## 2. Model Description

**Figure 1:**
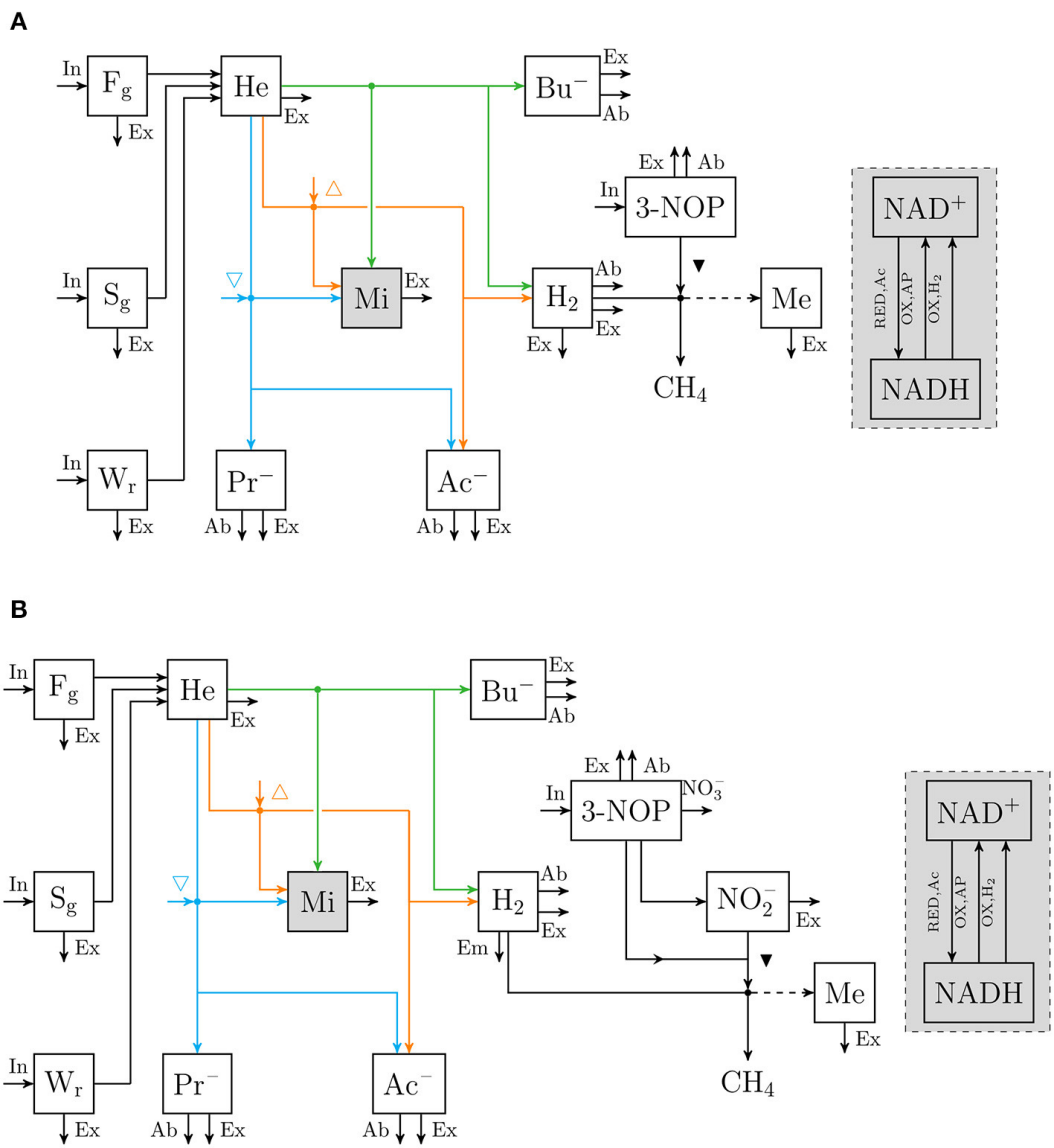
Flow chart that conceptually represent **(A)** the rumen 3-nitrooxypropanol simple model, and **(B)** the rumen 3-nitrooxypropanol+nitrite model. Boxes enclosed by solid lines represent state variables (with F_g_ for degradable fiber [g], S_g_ for degradable starch [g], W_r_ for soluble carbohydrates [g], He for hexose [mol], Mi for fermentative microbes [g], Ac^−^ for acetate [mol], Pr^−^ for propionate [mol], Bu^−^ for butyrate [mol], H_2_ for hydrogen [mol], 3-NOP for 3-nitrooxypropanol [mol], $\text{N}{\text{O}}_{2}^{-}$ for nitrite [mol], Me for methanogens [g]. The sum of NAD^+^ and NADH [mol] is a fraction of Mi and a gray fill is used to visualize this), arrows represent fluxes with the dashed arrow indicating H_2_ is not incorporated but its conversion to CH_4_ is required for growth (with In for dietary input, Ex for fractional exit from the rumen to the lower tract, Ab fractional absorption, Em for fractional emission, $\text{N}{\text{O}}_{3}^{-}$ for nitrate production, RED, Ac for NAD^+^ reduction associated with hexose converted into 2 Ac, {OX,AP} for NADH oxidation associated with hexose converted into $\frac{2}{3}$ Ac^−^ + $\frac{4}{3}$ Pr^−^, and {OX,H_2_} for hydrogenase catalyzed NADH oxidation; △ and ▽ indicate that at increased NAD^+^ to NADH ratio the microbial conversion is promoted and inhibited, respectively; ▾ indicates inhibition of methanogenesis; fluxes may be unique per state variable and are further specified in Van Lingen et al. (2019), dots indicate microbial conversions.

**Figure 2:**
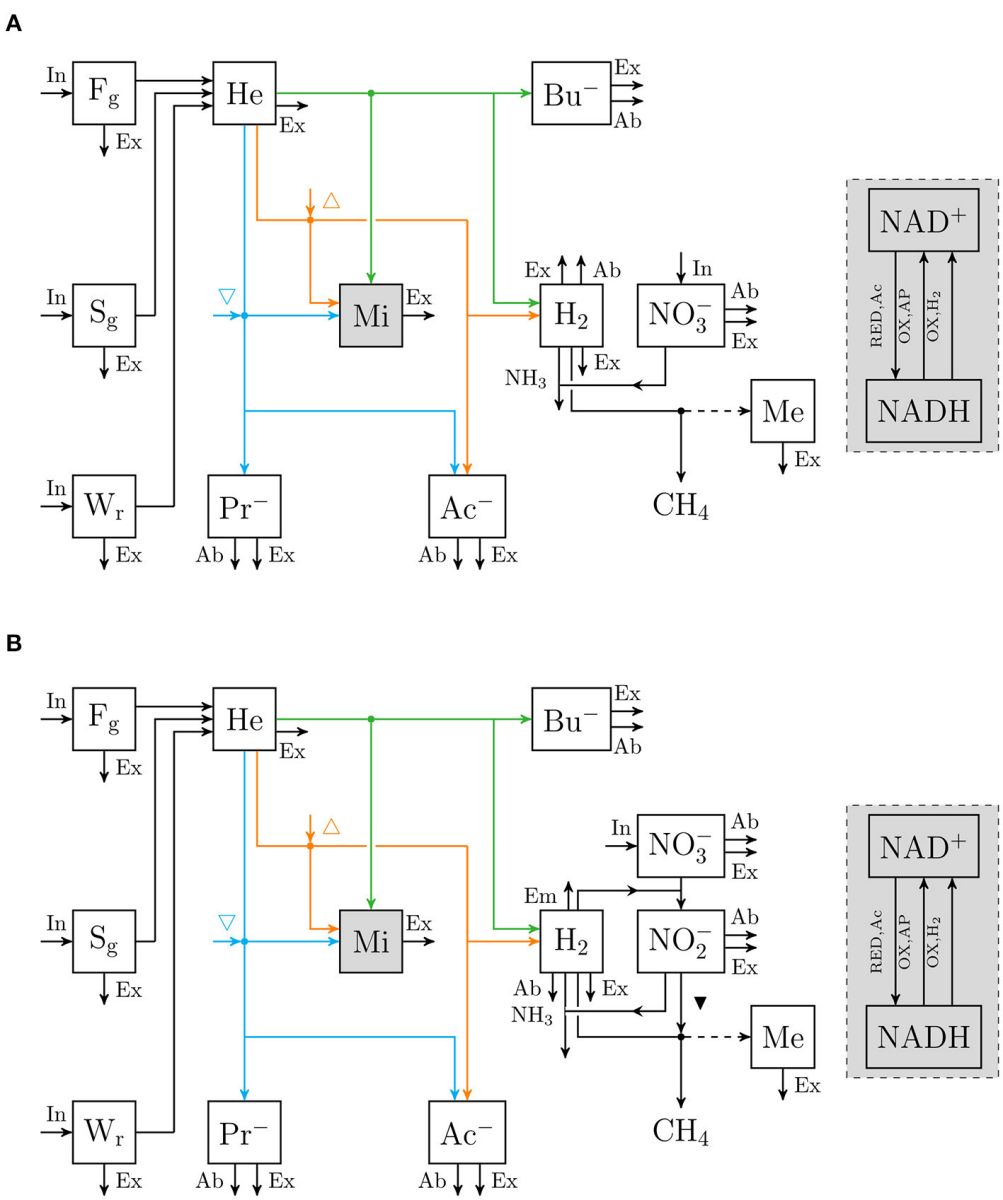
Flow chart that conceptually represent **(A)** the rumen nitrate simple model, and **(B)** the rumen nitrate+nitrite model. Boxes enclosed by solid lines represent state variables (with $\text{N}{\text{O}}_{3}^{-}$ for nitrate [mol] and $\text{N}{\text{O}}_{2}^{-}$ for nitrite [mol]; ▾ indicates inhibition of methanogenesis; other abbreviations are described in Figure 1), dots indicate microbial conversions.

An extant dynamic mechanistic rumen fermentation model with state variables for ruminal carbohydrate substrates, bacteria and protozoa, gaseous and dissolved fermentation end products and methanogens (Van Lingen et al., 2019) was extended with a representation of either the 3-NOP or $\text{N}{\text{O}}_{3}^{-}$ metabolism. The extant model represents the hydrolysis of carbohydrate polymers (viz., degradable fiber, degradable starch and sugars) into hexose, the thermodynamic control of *p*_H_2__ on volatile fatty acid (VFA) fermentation pathways via the NAD^+^ to NADH ratio in fermentative microbes, and hydrogenotrophic methanogenesis in the bovine rumen. Four different extensions of the original model were made. These model extensions comprised a representation of 3-NOP and $\text{N}{\text{O}}_{3}^{-}$ and with and without $\text{N}{\text{O}}_{2}^{-}$, which is derived from both 3-NOP and $\text{N}{\text{O}}_{3}^{-}$, respectively. The four extended models are diagrammatically represented in Figures 1, 2, while a schematic overview of physiological characteristics incorporated per model is provided in Table 1A. Mathematical notation of influxes and outfluxes of model state variables is *P*_i;j, m_ and *U*_i;j, m;n_, respectively, where the subscript represents the uptake or production of *i* by *j*-to-*m* transaction (generating *n*). To illustrate this, *P*_3NOP;In, 3NOP_ represents the increase in 3-NOP as a result of the inflow of 3-NOP. Concentrations of *i* are computed as:

(1) $$\begin{array}{l} C_{i}=\frac{Q_{i}}{V_{\text{Fl}}} \end{array}$$

for $i=\left\{{\text{H}}_{2},3-\text{NOP},\text{N}{\text{O}}_{3}^{-},\text{N}{\text{O}}_{2}^{-}\right\}$ and *V*_Fl_ being the rumen fluid volume. State variables are expressed in [g] or [mol], with the corresponding fluxes and concentrations expressed in [mol·h^−1^] or [g·h^−1^], and [mol·L^−1^] or [g·L^−1^], respectively. Abbreviations and general notation are available in Table 2. Parameters specific for the new models are provided in Table 3.

**Table 1:** Overview of **(A)** physiological characteristics regarding methanogenic inhibition and H_2_ sinks incorporated in 3-NOP, 3-NOP+nitrite, nitrate and nitrate+nitrite models, along with **(B)** the physiological response of various output variables to dietary inclusion of 3-NOP or $\text{N}{\text{O}}_{3}^{-}$.

	**3-NOP**	**3-NOP+nitrite**	**Nitrate**	**Nitrate+nitrite**
**(A) Physiological characteristic**				
Methanogenic inhibition by 3-NOP (MCR^a^)	✓	✓		
3-NOP to $\text{N}{\text{O}}_{3}^{-}$ + $\text{N}{\text{O}}_{2}^{-}$ (MCR^a^)		✓		
Methanogenic inhibition by $\text{N}{\text{O}}_{2}^{-}$ (MCR^a^)		✓		
$\text{N}{\text{O}}_{3}^{-}$ to NH_3_ (H_2_ sink)			✓	
$\text{N}{\text{O}}_{3}^{-}$ to $\text{N}{\text{O}}_{2}^{-}$ (H_2_ sink)				✓
$\text{N}{\text{O}}_{2}^{-}$ to NH_3_ (H_2_ sink)				✓
Methanogenic inhibition by $\text{N}{\text{O}}_{2}^{-}$ (hypothesized^b^)				✓
**(B) Response to supplemented 3-NOP or** $\text{N}{\text{O}}_{3}^{-}$				
H_2_ emission rate and *p*_H_2__	↑	↑	↓	↑
CH_4_ emission rate	↓	↓	↓	↓
Inhibition on NADH oxidation	↑	↑	↓	↑
Acetate proportion	↓	↓	-	↓
Propionate proportion	↑	↑	-	↑
Butyrate proportion	↑	↑	-	↑

*Check-marks indicate if a physiological characteristic was incorporated. Upward and downward arrows indicate up-regulation and down-regulation of the metabolism, respectively, a dash indicates a response was negligibly small*.

a *Reflects inhibition of archaeal methyl co-enzyme-M reductase*.

b *Mode of action of methanogenic inhibition by $\text{N}{\text{O}}_{3}^{-}$ derived $\text{N}{\text{O}}_{2}^{-}$ remains to be fully determined. See also sections 2.1.4 and 4.2*.

**Table 2:** Abbreviations used in mathematical expressions in the model.

**Symbol**	**Entity**
Ab	Absorption
Ac	Acetate
AP	Acetate + propionate
Bu	Butyrate
DM	Dry matter
Em	Emission (from the rumen)
Ex	Exit to lower tract
F_g_	Degradable fiber
Fl	Fluid
He	Hexose
In	Intake
La	Lactate
Me	Methanogens
Mi	Fermentative microbes
P_g_	Degradable protein
Pr	Propionate
P_s_	Soluble protein
S_g_	Degradable starch
So	Solid
S_r_	Soluble starch
W_r_	Water soluble carbohydrates

**Table 3:** Preliminary parameter values used in the 3-NOP, 3-NOP+$\text{N}{\text{O}}_{2}^{-}$, $\text{N}{\text{O}}_{3}^{-}$, and $\text{N}{\text{O}}_{3}^{-}$+$\text{N}{\text{O}}_{2}^{-}$ models.

**Variable**	**Units**	**3-NOP**	$3-NO\text{P}+{\text{N}\text{O}}_{2}^{-}$	${\text{N}\text{O}}_{3}^{-}$	${\text{N}\text{O}}_{3}^{-}+{\text{N}\text{O}}_{2}^{-}$
$k_{\text{N}{\text{O}}_{\text{x}}^{-},\text{Ab}}$	h^−1^	–	–	0.30	0.30
*k*_3NOP,Ab_	h^−1^	0.30	0.30	–	–
$k_{\text{N}{\text{O}}_{3}^{-},\text{N}{\text{H}}_{3}}$	mol^−1^g^−1^h^−1^			6.99	
$k_{\text{N}{\text{O}}_{3}^{-},\text{N}{\text{O}}_{2}^{-}}$	mol^−1^g^−1^h^−1^				1.5
$k_{\text{N}{\text{O}}_{2}^{-},\text{N}{\text{H}}_{3}}$	mol^−1^g^−1^h^−1^				0.113
$k_{3\text{NOP},\text{N}{\text{O}}_{3}^{-}}$	g^−1^h^−1^		1.55		
$k_{3\text{NOP},\text{N}{\text{O}}_{2}^{-}}$	g^−1^h^−1^		0.44		
$J_{\text{N}{\text{O}}_{2}^{-};{\text{H}}_{2},\text{Me}}$	M				1.17e-3
*J*_MCR,_H__2_, Me_	M	1.93e-5	2.10e-5		

### 2.1. Mathematical Representation of Model Extentions

#### 2.1.1. 3-NOP Simple Model

3-nitrooxypropanol state variable, *Q*_3NOP_ [mol]. The *Q*_3NOP_ state variable receives input from 3-NOP contents in the feed that was supplemented:

(2) $$\begin{array}{l} P_{3\text{NOP};\text{In},3\text{NOP}}=D_{\text{DM}}\left(t\right)\cdot c_{3\text{NOP}} \end{array}$$

with *D*_DM_(*t*) the dry matter intake rate in time [kg·h^−1^] and *c*_3NOP_ the 3-NOP content of the feed [mol·kg^−1^]. 3-NOP can easily diffuse through membranes (Duin et al., 2016) and was assumed to be absorbed across the rumen wall:

(3) $$\begin{array}{l} U_{3\text{NOP};3\text{NOP},\text{Ab}}=k_{3\text{NOP},\text{Ab}}\cdot Q_{3\text{NOP}}, \end{array}$$

with *k*_3NOP,Ab_ the fractional absorption rate of 3-NOP (value and units in Table 3). Finally 3-NOP was assumed to flow out to the lower tract with the fluid fraction, which was represented as:

(4) $$\begin{array}{l} U_{3\text{NOP};3\text{NOP},\text{Ex}}=k_{\text{Fl},\text{Ex}}\cdot Q_{3\text{NOP}} \end{array}$$

with *k*_Fl,Ex_ the fractional outflow rate of the fluid fraction [h^−1^] as in Van Lingen et al. (2019). The differential equation of the *Q*_3NOP_ state variable is given by:

(5) $$\begin{array}{l} \frac{\text{d}Q_{3\text{NOP}}}{\text{d}t}=P_{3\text{NOP};\text{In},3\text{NOP}}-U_{3\text{NOP};3\text{NOP},\text{Ab}}-U_{3\text{NOP};3\text{NOP},\text{Ex}} \end{array}$$

Hydrogen state variable, *Q*_H_2__ [mol]. As described by Van Lingen et al. (2019), inputs to the *Q*_H_2__ state variable are H_2_ influxes associated with acetate and butyrate production (*P*_H_2_; He, Ac_ and *P*_H_2_; He, Bu_), whereas outputs that are copied to the present model are emission, outflow with rumen fluid and absorption of H_2_ (*U*_H_2_; H_2_, Em_, *U*_H_2_; H_2_, Ex_, and *U*_H_2_; H_2_, Ab_, respectively). In the present model, the outflux that represents H_2_ utilization for 3-NOP inhibited methanogenic growth is given by:

(6) $$\begin{array}{l} U_{{\text{H}}_{2};{\text{H}}_{2},\text{C}{\text{H}}_{4}}=\frac{v_{{\text{H}}_{2},\text{C}{\text{H}}_{4}}\cdot Q_{\text{Me}}}{1+\frac{M_{{\text{H}}_{2};{\text{H}}_{2},\text{C}{\text{H}}_{4}}}{C_{{\text{H}}_{2}}}+\frac{C_{3\text{NOP}}}{J_{\text{MCR};{\text{H}}_{2},\text{C}{\text{H}}_{4}}}} \end{array}$$

where *v*_H_2_, CH_4__ denotes the maximum utilization rate of H_2_ by archaea [mol·g^−1^h^−1^; from Van Lingen et al. (2019)], *Q*_Me_ the methanogen state variable, *M*_H_2_; H_2_, CH_4__ the saturation constant for H_2_ utilization for methangenesis [M; from Van Lingen et al. (2019)], *C*_H_2__ the dissolved H_2_ concentration, *C*_3NOP_ the 3-NOP concentration and *J*_MCR;_H__2_, CH_4__ the inhibition constant of 3-NOP associated with hydrogenotrophic methanogenesis (Table 3). The differential equation is given by:

(7) $$\begin{array}{l} \frac{\text{d}Q_{{\text{H}}_{2}}}{\text{d}t}=P_{{\text{H}}_{2};\text{He},\text{Ac}}-P_{{\text{H}}_{2};\text{He},\text{Bu}}-U_{{\text{H}}_{2};{\text{H}}_{2},\text{Ex}}-U_{{\text{H}}_{2};{\text{H}}_{2},\text{Em}}\\ \;-U_{{\text{H}}_{2};{\text{H}}_{2},\text{Ab}}-U_{{\text{H}}_{2};{\text{H}}_{2},\text{C}{\text{H}}_{4}.} \end{array}$$

#### 2.1.2. 3-Nitrooxypropanol+Nitrite Model

According to Duin et al. (2016), 3-NOP is broken down to $\text{N}{\text{O}}_{3}^{-}$ and $\text{N}{\text{O}}_{2}^{-}$ along with the formation of 1,3-propanediol. These conversions may take place in the archaeal cytosol that contribute to the presence $\text{N}{\text{O}}_{2}^{-}$ that also inhibits MCR. For evaluating the implications of these metabolic steps, an extended 3-NOP model was developed that also comprised a $Q_{\text{N}{\text{O}}_{2}^{-}}$ state variable.

3-nitrooxypropanol state variable, *Q*_3NOP_ [mol]. In addition to the inputs and outputs described for the simple 3-NOP model, the conversion of 3-NOP into $\text{N}{\text{O}}_{3}^{-}$ and $\text{N}{\text{O}}_{2}^{-}$ is described as output from the *Q*_3NOP_ state variable in the present model by:

(8) $$U_{\text{3NOP};\text{3NOP},{\text{NO}}_{3}^{-}}=k_{\text{3NOP},{\text{NO}}_{3}^{-}}\cdot Q_{\text{Me}}\cdot Q_{\text{3NOP}}$$

and

(9) $$U_{\text{3NOP};\text{3NOP},{\text{NO}}_{2}^{-}}=k_{\text{3NOP},{\text{NO}}_{2}^{-}}\cdot Q_{\text{Me}}\cdot Q_{\text{3NOP}}$$

swith $k_{3\text{NOP},\text{N}{\text{O}}_{3}^{-}}$ and $k_{3\text{NOP},\text{N}{\text{O}}_{2}^{-}}$ the fractional rate constants for the conversion of 3-NOP reduction to $\text{N}{\text{O}}_{3}^{-}$ and $\text{N}{\text{O}}_{2}^{-}$ (Table 3) and the reduction flow rate is assumed to be also dependent on the methanogenic biomass. It was assumed that $\text{N}{\text{O}}_{3}^{-}$ and $\text{N}{\text{O}}_{2}^{-}$ is not transported across the methanogenic cell membrane and no other outputs were represented. This resulted in the differential equation of the *Q*_3NOP_ state variable in the 3-NOP extended model given by:

(10) $$\begin{array}{l} \frac{\text{d}Q_{\text{3NOP}}}{\text{d}t}=P_{\text{3NOP};\text{In},\text{3NOP}}-U_{\text{3NOP};\text{3NOP},{\text{NO}}_{3}^{-}}-U_{\text{3NOP};\text{3NOP},{\text{NO}}_{2}^{-}}\\ \;-U_{\text{3NOP};\text{3NOP},\text{Ab}}-U_{\text{3NOP};\text{3NOP},\text{Ex}} \end{array}$$

Nitrite state variable, $Q_{\text{N}{\text{O}}_{2}^{-}}$ [mol]. Input to the $Q_{\text{N}{\text{O}}_{2}^{-}}$ state variable was $\text{N}{\text{O}}_{2}^{-}$ production from 3-NOP reduction:

(11) $$P_{{\text{NO}}_{2}^{-};\text{3NOP},{\text{NO}}_{2}^{-}}=U_{\text{3NOP};\text{3NOP},{\text{NO}}_{2}^{-}}$$

and outflow from the rumen to the lower tract is with the methanogens as in Van Lingen et al. (2019):

(12) $$U_{{\text{NO}}_{2}^{-};{\text{NO}}_{2}^{-},\text{Ex}}=0.4\cdot (k_{\text{Fl},\text{Ex}}+k_{\text{So},\text{Ex}})\cdot Q_{{\text{NO}}_{2}^{-}}$$

with *k*_So,Ex_ the fractional outflow rate of the solid material as in Van Lingen et al. (2019). The differential equation is given by:

(13) $$\frac{\text{d}Q_{{\text{NO}}_{2}^{-}}}{\text{d}t}=P_{{\text{NO}}_{2}^{-};\text{3NOP},{\text{NO}}_{2}^{-}}-U_{{\text{NO}}_{2}^{-};{\text{NO}}_{2}^{-},\text{Ex}}$$

Hydrogen state variable, *Q*_H_2__ [mol]. Compared with the 3-NOP simple model, the outflux that represents H_2_ utilization for methanogenesis in the 3-NOP+nitrite model also accounts for inhibition of methanogenic growth by $\text{N}{\text{O}}_{2}^{-}$, which is given by:

(14) $$U_{{\text{H}}_{2};{\text{H}}_{2},{\text{CH}}_{4}}=\frac{v_{{\text{H}}_{2},{\text{CH}}_{4}}\cdot Q_{\text{Me}}}{1+\frac{M_{{\text{H}}_{2};{\text{H}}_{2},{\text{CH}}_{4}}}{C_{{\text{H}}_{2}}}+\frac{C_{\text{3NOP}}+C_{{\text{NO}}_{2}^{-}}}{J_{\text{MCR};{\text{H}}_{2},{\text{CH}}_{4}}}}$$

where *J*_MCR;_H__2_, CH_4__ denotes the inhibition constant with respect to the aggregated concentrations of 3-NOP and $\text{N}{\text{O}}_{2}^{-}$ (Table 3). The differential equation for the 3-NOP+$\text{N}{\text{O}}_{2}^{-}$ extended model is given by:

(15) $$\begin{array}{l} \frac{\text{d}Q_{{\text{H}}_{2}}}{\text{d}t}=P_{{\text{H}}_{2};\text{He},\text{Ac}}-P_{{\text{H}}_{2};\text{He},\text{Bu}}-U_{{\text{H}}_{2};{\text{H}}_{2},\text{Ex}}\\ \;-U_{{\text{H}}_{2};{\text{H}}_{2},\text{Em}}-U_{{\text{H}}_{2};{\text{H}}_{2},\text{Ab}}-U_{{\text{H}}_{2};{\text{H}}_{2},\text{C}{\text{H}}_{4}.} \end{array}$$

#### 2.1.3. Nitrate Simple Model

The key mechanism for the decrease in CH_4_ production after supplementing $\text{N}{\text{O}}_{3}^{-}$ is generally considered the utilization of H_2_ (Yang et al., 2016). The model was extended with only a $\text{N}{\text{O}}_{3}^{-}$ state variable for evaluating the significance of this mechanism.

Nitrate state variable, $Q_{\text{N}{\text{O}}_{3}^{-}}$ [mol]. The $Q_{\text{N}{\text{O}}_{3}^{-}}$ state variable receives input from $\text{N}{\text{O}}_{3}^{-}$ contents in the feed that was supplemented:

(16) $$P_{{\text{NO}}_{3}^{-};\text{In},{\text{NO}}_{3}^{-}}=D_{\text{DM}}(t)\cdot c_{{\text{NO}}_{3}^{-}}$$

with $c_{\text{N}{\text{O}}_{3}^{-}}$ the $\text{N}{\text{O}}_{3}^{-}$ content of the feed [mol·kg^−1^]. Output comprised the reduction of $\text{N}{\text{O}}_{3}^{-}$ to NH_3_ in the periplasm of fermentative microbes (Kern and Simon, 2009):

(17) $$U_{{\text{NO}}_{3}^{-};{\text{NO}}_{3}^{-},{\text{NH}}_{3}}=k_{{\text{NO}}_{3}^{-},{\text{NH}}_{3}}\cdot Q_{\text{Mi}}\cdot Q_{{\text{NO}}_{3}^{-}}\cdot Q_{{\text{H}}_{2}}$$

with $k_{\text{N}{\text{O}}_{3}^{-},\text{N}{\text{H}}_{3}}$ the rate constant for $\text{N}{\text{O}}_{3}^{-}$ reduction to NH_3_ (Table 3). The absorption of $\text{N}{\text{O}}_{3}^{-}$ across the rumen wall was represented as:

(18) $$U_{{\text{NO}}_{3}^{-};{\text{NO}}_{3}^{-},\text{Ab}}=k_{{\text{NO}}_{\text{x}}^{-},\text{Ab}}\cdot Q_{{\text{NO}}_{3}^{-}}$$

with $k_{\text{N}{\text{O}}_{\text{x}}^{-},\text{Ab}}$ the fractional absorption rate for $\text{N}{\text{O}}_{3}^{-}$ absorption (Table 3). $\text{N}{\text{O}}_{3}^{-}$ was assumed to flow out with the fluid fraction from the rumen to the lower tract:

(19) $$U_{{\text{NO}}_{3}^{-};{\text{NO}}_{3}^{-},\text{Ex}}=k_{\text{Fl},\text{Ex}}\cdot Q_{{\text{NO}}_{3}^{-}}$$

The differential equation is given by:

(20) $$\begin{array}{l} \frac{\text{d}Q_{{\text{NO}}_{3}^{-}}}{\text{d}t}=P_{{\text{NO}}_{3}^{-};\text{In},{\text{NO}}_{3}^{-}}-U_{{\text{NO}}_{3}^{-};{\text{NO}}_{3}^{-},\text{Ab}}\\ \;-U_{{\text{NO}}_{3}^{-};{\text{NO}}_{3}^{-},\text{Ex}}-U_{{\text{NO}}_{3}^{-};{\text{NO}}_{3}^{-},{\text{NH}}_{3}} \end{array}$$

Hydrogen state variable, *Q*_H_2__ [mol]. Influxes and outfluxes that were taken from Van Lingen et al. (2019) were the same as for the 3-NOP model. In the $\text{N}{\text{O}}_{3}^{-}$ model, output represented H_2_ utilization for $\text{N}{\text{O}}_{3}^{-}$ reduction to NH_3_ while applying a 4:1 stoichiometric ratio:

(21) $$U_{{\text{H}}_{2};{\text{NO}}_{3}^{-},{\text{NH}}_{3}}=4\cdot U_{{\text{NO}}_{3}^{-};{\text{NO}}_{3}^{-},{\text{NH}}_{3}}$$

The flux that represented H_2_ utilization for methanogenic growth was copied from the Van Lingen et al. (2019) model:

(22) $$\begin{array}{r} U_{{\text{H}}_{2};{\text{H}}_{2},CH_{4}}=\frac{v_{{\text{H}}_{2},\text{C}{\text{H}}_{4}}\cdot Q_{\text{Me}}}{1+\frac{M_{{\text{H}}_{2};{\text{H}}_{2},\text{C}{\text{H}}_{4}}}{C_{{\text{H}}_{2}}}} \end{array}$$

The differential equation is given by:

(23) $$\begin{array}{l} \frac{\text{d}Q_{{\text{H}}_{2}}}{\text{d}t}=P_{{\text{H}}_{2};\text{He},\text{Ac}}+P_{{\text{H}}_{2};\text{He},\text{Bu}}-U_{{\text{H}}_{2};{\text{H}}_{2},\text{Me}}-U_{{\text{H}}_{2};{\text{NO}}_{3}^{-},{\text{NH}}_{3}}\\ \;-U_{{\text{H}}_{2};{\text{H}}_{2},\text{Ab}}-U_{{\text{H}}_{2};{\text{H}}_{2},\text{Em}}-U_{{\text{H}}_{2};{\text{H}}_{2},\text{Ex}.} \end{array}$$

#### 2.1.4. Nitrate+Nitrite Model

For evaluating the significance of the $\text{N}{\text{O}}_{2}^{-}$ intermediary metabolite on the metabolism, an extended $\text{N}{\text{O}}_{3}^{-}$ model was developed for which a $Q_{\text{N}{\text{O}}_{2}^{-}}$ state variable was also included.

Nitrate state variable, $Q_{\text{N}{\text{O}}_{3}^{-}}$ [mol]. The $U_{\text{N}{\text{O}}_{3}^{-};\text{N}{\text{O}}_{3}^{-},\text{N}{\text{H}}_{3}}$ of the $Q_{\text{N}{\text{O}}_{3}^{-}}$ state variable in the simple model was broken up in two parts in the extended model. The first part resulted in output that comprised the reduction of $\text{N}{\text{O}}_{3}^{-}$ to $\text{N}{\text{O}}_{2}^{-}$ in the periplasm of fermentative microbes (Kern and Simon, 2009):

(24) $$U_{{\text{NO}}_{3}^{-};{\text{NO}}_{3}^{-},{\text{NO}}_{2}^{-}}=k_{NO_{3}^{-},NO_{2}^{-}}\cdot Q_{\text{Mi}}\cdot Q_{NO_{3}^{-}}\cdot Q_{{\text{H}}_{2}}$$

with $k_{\text{N}{\text{O}}_{3}^{-},\text{N}{\text{O}}_{2}^{-}}$ the rate constant for $\text{N}{\text{O}}_{3}^{-}$ reduction to $\text{N}{\text{O}}_{2}^{-}$ by fermentative microbes (Table 3). Inflow, absorption across the rumen wall and outflow to the lower gastrointestinal tract were represented identical to the nitrate simple model, which resulted in a differential equation given by:

(25) $$\begin{array}{l} \frac{\text{d}Q_{{\text{NO}}_{3}^{-}}}{\text{d}t}=P_{{\text{NO}}_{3}^{-};\text{In},{\text{NO}}_{3}^{-}}-U_{{\text{NO}}_{3}^{-};{\text{NO}}_{3}^{-},\text{Ab}}-U_{{\text{NO}}_{3}^{-};{\text{NO}}_{3}^{-},\text{Ex}}\\ \;-U_{{\text{NO}}_{3}^{-};{\text{NO}}_{3}^{-},{\text{NO}}_{2}^{-}} \end{array}$$

Nitrite state variable, $Q_{\text{N}{\text{O}}_{2}^{-}}$ [mol]. Input to the $Q_{\text{N}{\text{O}}_{2}^{-}}$ state variable was $\text{N}{\text{O}}_{2}^{-}$ production from $\text{N}{\text{O}}_{3}^{-}$ reduction:

(26) $$P_{{\text{NO}}_{2}^{-};{\text{NO}}_{3}^{-},{\text{NO}}_{2}^{-}}=U_{{\text{NO}}_{3}^{-};{\text{NO}}_{3}^{-},{\text{NO}}_{2}^{-}},$$

whereas output from this state variable comprised absorption of $\text{N}{\text{O}}_{2}^{-}$ across the rumen wall:

(27) $$U_{{\text{NO}}_{2}^{-};{\text{NO}}_{2}^{-},\text{Ab}}=k_{{\text{NO}}_{\text{x}}^{-},\text{Ab}}\cdot Q_{{\text{NO}}_{2}^{-}}$$

with $k_{\text{N}{\text{O}}_{\text{x}}^{-},\text{Ab}}$ the fractional absorption rate for $\text{N}{\text{O}}_{2}^{-}$, which was also used for $\text{N}{\text{O}}_{3}^{-}$ absorption. The outflow of $\text{N}{\text{O}}_{2}^{-}$ was with the fluid fraction from the rumen to the lower tract:

(28) $$U_{{\text{NO}}_{2}^{-};{\text{NO}}_{2}^{-},\text{Ex}}=k_{\text{Fl},\text{Ex}}\cdot Q_{{\text{NO}}_{2}^{-}}$$

and the reduction of $\text{N}{\text{O}}_{2}^{-}$ to NH_3_:

(29) $$U_{{\text{NO}}_{2}^{-};{\text{NO}}_{2}^{-},{\text{NH}}_{3}}=k_{{\text{NO}}_{2}^{-},{\text{NH}}_{3}}\cdot Q_{\text{Mi}}\cdot Q_{{\text{NO}}_{2}^{-}}\cdot Q_{{\text{H}}_{2}}$$

where $k_{\text{N}{\text{O}}_{2}^{-},\text{N}{\text{H}}_{3}}$ denotes the rate constant for $\text{N}{\text{O}}_{2}^{-}$ reduction to NH_3_ by fermentative microbes (Table 3). The differential equation is given by:

(30) $$\begin{array}{l} \frac{\text{d}Q_{{\text{NO}}_{2}^{-}}}{\text{d}t}=P_{{\text{NO}}_{2}^{-};{\text{NO}}_{3}^{-},{\text{NO}}_{2}^{-}}-U_{{\text{NO}}_{2}^{-};{\text{NO}}_{2}^{-},\text{Ab}}\\ \;-U_{{\text{NO}}_{2}^{-};{\text{NO}}_{2}^{-},\text{Ex}}-U_{{\text{NO}}_{2}^{-};{\text{NO}}_{2}^{-},{\text{NH}}_{3}} \end{array}$$

Hydrogen state variable, *Q*_H_2__ [mol]. Influxes and outfluxes that were taken from Van Lingen et al. (2019) were the same as for the 3-NOP models and the reduced $\text{N}{\text{O}}_{3}^{-}$ model. In the full $\text{N}{\text{O}}_{3}^{-}$ model, output represented H_2_ utilization for $\text{N}{\text{O}}_{3}^{-}$ reduction to $\text{N}{\text{O}}_{2}^{-}$ while applying a 1:1 stoichiometric ratio:

(31) $$U_{{\text{H}}_{2};{\text{NO}}_{3}^{-},{\text{NO}}_{2}^{-}}=U_{{\text{NO}}_{3}^{-};{\text{NO}}_{3}^{-},{\text{NO}}_{2}^{-}}$$

and H_2_ utilization for $\text{N}{\text{O}}_{2}^{-}$ reduction to NH_3_ while applying a 3:1 stoichiometric ratio:

(32) $$U_{{\text{H}}_{2};{\text{NO}}_{2}^{-},{\text{NH}}_{3}}=3\cdot U_{{\text{NO}}_{2}^{-};{\text{NO}}_{2}^{-},{\text{NH}}_{3}}$$

Rumen methanogens without cytochromes were suggested to be inhibited by $\text{N}{\text{O}}_{2}^{-}$ (Latham et al., 2016) at their electron-carrier system (Yang et al., 2016). Therefore, the flux that represented H_2_ utilization for methanogenic growth that was incorporated accounted for inhibition by $\text{N}{\text{O}}_{2}^{-}$:

(33) $$U_{{\text{H}}_{2};{\text{H}}_{2},\text{C}{\text{H}}_{4}}=\frac{v_{{\text{H}}_{2},\text{C}{\text{H}}_{4}}\cdot Q_{\text{Me}}}{1+\frac{M_{{\text{H}}_{2};{\text{H}}_{2},\text{C}{\text{H}}_{4}}}{C_{{\text{H}}_{2}}}+\frac{C_{{\text{NO}}_{2}^{-}}}{J_{{\text{NO}}_{2}^{-};{\text{H}}_{2},{\text{CH}}_{4}}}}$$

where $C_{\text{N}{\text{O}}_{2}^{-}}$ denotes the H_2_ concentration, $J_{\text{N}{\text{O}}_{2}^{-};{\text{H}}_{2},\text{C}{\text{H}}_{4}}$ the inhibition constant for $\text{N}{\text{O}}_{2}^{-}$ of the H_2_ uptake rate for methanogenesis (Table 3). The differential equation is given by:

(34) $$\begin{array}{l} \frac{\text{d}Q_{{\text{H}}_{2}}}{\text{d}t}=P_{{\text{H}}_{2};\text{He},\text{Ac}}+P_{{\text{H}}_{2};\text{He},\text{Bu}}-U_{{\text{H}}_{2};{\text{H}}_{2},\text{Me}}-U_{{\text{H}}_{2};{\text{NO}}_{3}^{-},{\text{NO}}_{2}^{-}}\\ \;-U_{{\text{H}}_{2};{\text{NO}}_{2}^{-},{\text{NH}}_{3}}-U_{{\text{H}}_{2};{\text{H}}_{2},\text{Em}}-U_{{\text{H}}_{2};{\text{H}}_{2},\text{Ab}}-U_{{\text{H}}_{2};{\text{H}}_{2},\text{Ex}.} \end{array}$$

### 2.2. Model Input and Parameter Values

Inputs to the model were intake rate (shown in Figures 1, 2) and nutrient composition of DM (Table 4). These inputs were taken from Van Zijderveld et al. (2011), Veneman et al. (2015), and Olijhoek et al. (2016) for the $\text{N}{\text{O}}_{3}^{-}$ models, whereas the inputs were taken from Haisan et al. (2014), Hristov et al. (2015), Lopes et al. (2016), Haisan et al. (2017), and Van Wesemael et al. (2019) for the 3-NOP models. Every simulation was based on a dietary treatment with the inclusion rates of 3-NOP and $\text{N}{\text{O}}_{3}^{-}$ that was supplemented. If the feed intake rate in time was not reported, feed intake rates were scaled to Olijhoek et al. (2016) for *ad libitum* feeding and scaled to Van Lingen et al. (2017) for restricted feeding. This scaling was done based on the fraction of daily feed intake consumed per hour of a day. The dietary nutrient contents and *k*_FgHe_ and *k*_SgHe_ for the different studies were set per dietary treatment and taken in accordance with Bannink et al. (2010) and CVB (2018). Non-identified fractions that may include pectin and fructan were assigned to Fg, Sg, and Wr as in Van Lingen et al. (2019). An overview of all nutrient contents and degradation characteristics is given in Table 4. For evaluating the biological significance of 3-NOP and $\text{N}{\text{O}}_{3}^{-}$ on the rumen microbial metabolism, the 3-NOP models were run for supplement inclusion rates of 0, 0.5, and 1.0 mmol·(kg DMI)^−1^, whereas the $\text{N}{\text{O}}_{3}^{-}$ models were run for inclusion rates of 0, 0.16, and 0.32 mol·(kg DMI)^−1^. Dry matter intake rate and composition input data were from Van Lingen et al. (2017) on which various parameters of the extant model were fitted previously.

**Table 4:** Degradable fiber (Fg), degradable starch (Sg), degradable protein (Pg) soluble sugars (Wr), acetate (Ac^−^), propionate (Pr^−^), butyrate (Bu^−^), and lactate (La^−^) feed contents [g·kg^−1^], and fractional hydrolysis rates [h^−1^] of degradable fiber and degradable starch per experiment and/or treatment assigned (ExpTr) for 3-NOP and $\text{N}{\text{O}}_{3}^{-}$ model fitting data from Olijhoek et al. (2016, O), Van Zijderveld et al. (2011, VZ), Veneman et al. (2015, VM), Haisan et al. (2014, Hn1), Hristov et al. (2015, Hv), Haisan et al. (2017, Hn2), Lopes et al. (2016, Ls) and Van Wesemael et al. (2019, VW), and model evaluation data from Van Lingen et al. (2017, VL, average across all treatments and cows).

**ExpTr**	**Fg**	**Sg**	**Wr**	**Ac^-^**	**Pr^-^**	**Bu^-^**	**La^-^**	***k*_FgHe_**	***k*_SgHe_**	***k*_PgPs_**
Data for fitting $\text{N}{\text{O}}_{3}^{-}$ models										
O	329	207	49	10	2	2	20	0.036	0.100	0.044
VZ	245	252	76	6	1	1	11	0.029	0.094	0.055
VM	294	230	90	13	2	2	26	0.025	0.100	0.054
Data for fitting 3-NOP models										
Hn1	281	250	140	0	0	0	0	0.056	0.091	0.067
Hv	198	244	157	3	0	0	5	0.043	0.087	0.070
Hn2	322	230	118	0	0	0	0	0.049	0.085	0.071
Ls	193	257	151	3	0	0	5	0.045	0.087	0.065
VW	315	149	116	6	1	1	12	0.042	0.081	0.053
Data for model evaluation										
VL	287	159	125	11	2	2	21	0.043	0.078	0.054

The differential equations of all state variables were numerically integrated for a given set of initial conditions and parameter values. The equations were solved using the lsoda numerical integration method (Petzold, 1983), a robust implicit integrator for stiff and non-stiff systems. This numerical integrator changes step size automatically to minimize computation time while maintaining calculation accuracy. The DM intake profile caused dramatic changes in *Q*_H_2__ shortly after feeding, which is why integration steps sizes were 2.5×10^−3^ h during the first 0.5 h and 10^−2^ h during the remaining hours of every consecutive 12 h period. Based on the absorption rate of $\text{N}{\text{O}}_{3}^{-}$ and $\text{N}{\text{O}}_{2}^{-}$ that was discussed to be slowly (Nolan et al., 2016), the $k_{\text{N}{\text{O}}_{\text{x}}^{-},\text{Ab}}$ parameter was assigned a value of 0.30 h^−1^, which is slightly lower than used for NH_3_ and VFA absorption in the Dijkstra et al. (1996) model. Given the lack of data on 3-NOP absorption, the same value was used for the *k*_3NOP,Ab_ parameter. Simulations based on the aforementioned collection of literature data were used for estimating the *J*_MCR;_H__2_, Me_ and $k_{\text{N}{\text{O}}_{3}^{-},\text{N}{\text{H}}_{3}}$ parameters of both 3-NOP models and the $\text{N}{\text{O}}_{3}^{-}$ model to average daily CH_4_ emission output. The $k_{\text{N}{\text{O}}_{2}^{-},\text{N}{\text{H}}_{3}}$ and $J_{\text{N}{\text{O}}_{2}^{-};{\text{H}}_{2},\text{Me}}$ parameters of the $\text{N}{\text{O}}_{3}^{-}$+$\text{N}{\text{O}}_{2}^{-}$ model were estimated to the diurnal H_2_ and CH_4_ emission rates that were extracted from the graphs presented in Van Zijderveld et al. (2011), Veneman et al. (2015) and Olijhoek et al. (2016). Including the $k_{3\text{NOP},\text{N}{\text{O}}_{3}^{-}}$, $k_{3\text{NOP},\text{N}{\text{O}}_{2}^{-}}$, and $k_{\text{N}{\text{O}}_{3}^{-},\text{N}{\text{O}}_{2}^{-}}$ in the parameter estimation procedure resulted in limited identifiability and these three parameters were assigned values more arbitrarily, but such that $\text{N}{\text{O}}_{2}^{-}$ concentrations in the 3-NOP+nitrite and nitrate+nitrate models approached the order of magnitude of the 3-NOP and $\text{N}{\text{O}}_{3}^{-}$ concentrations, respectively.

To avoid numerical dispersion during the parameter estimation procedure and to correct for the model inaccuracy, the model was run using control treatment input (i.e., no supplementation of 3-NOP and $\text{N}{\text{O}}_{3}^{-}$) for every study, after which the observed CH_4_ emission data for all dietary treatments for which a certain dose of 3-NOP and $\text{N}{\text{O}}_{3}^{-}$ was administered were multiplied by the ratio of the observed and predicted values. A 240 h run of the model was considered to have converged to quasi steady-state. Model output of the final 24 h vs. the experimental data were calculated to assess the model performance given the model parameter values. The parameters were optimized to minimize the sum of squared residuals values using the BFGS algorithm (Conn et al., 1991).

### 2.3. Global Sensitivity Analysis

The sensitivity of the CH_4_ emission rate to the parameters directly related to the inhibition was evaluated using a global sensitivity analysis. For this evaluation, the *J*_MCR;_H__2_, CH_4__, $J_{\text{N}{\text{O}}_{2}^{-};{\text{H}}_{2},\text{C}{\text{H}}_{4}}$, $k_{\text{N}{\text{O}}_{3}^{-},\text{N}{\text{O}}_{2}^{-}}$, $k_{\text{N}{\text{O}}_{2}^{-},\text{N}{\text{H}}_{3}}$, $k_{3\text{NOP},\text{N}{\text{O}}_{3}^{-}}$, $k_{3\text{NOP},\text{N}{\text{O}}_{2}^{-}}$, $k_{\text{N}{\text{O}}_{\text{x}}^{-},\text{Ab}}$, and *k*_3NOP,Ab_ parameters of the 3-NOP+$\text{N}{\text{O}}_{2}^{-}$ and $\text{N}{\text{O}}_{3}^{-}$+$\text{N}{\text{O}}_{2}^{-}$ models drawn from 0.75 to 1.25 times their optimum value using Latin hypercube sampling and a sample size of 1.000. The sensitivity of CH_4_ production was evaluated using the highest inclusion rates of 3-NOP and $\text{N}{\text{O}}_{3}^{-}$ and the Van Lingen et al. (2017) feed input. Correlation coefficients were calculated to quantify the sensitivity of the CH_4_ emission rate to the parameter values at 0, 0.5, 1, 2, 4, 6, and 10 h from the last meal of a 240 h simulation. All analyses were performed using the base (R Core Team, 2020) and FME packages (Soetaert and Petzoldt, 2010) in R statistical software.

## 3. Results

### 3.1. Models Solutions

**Figure 3:**
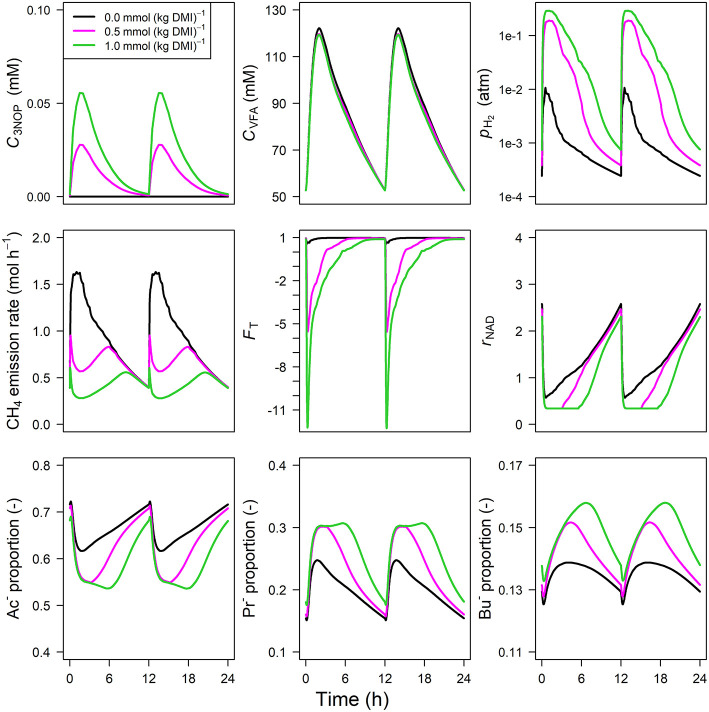
Solutions of the 3-NOP dynamic model without $\text{N}{\text{O}}_{2}^{-}$ representation [mM], VFA concentration [mM], rumen headspace *p*_H_2__ [atm], CH_4_ emission rates [mol·h^−1^], thermodynamic potential factor (*F*_T_; [–]), NAD^+^ to NADH ratio (*r*_NAD_), acetate proportion (Ac^−^), propionate proportion (Pr^−^), and butyrate proportion (Bu^−^).

**Figure 4:**
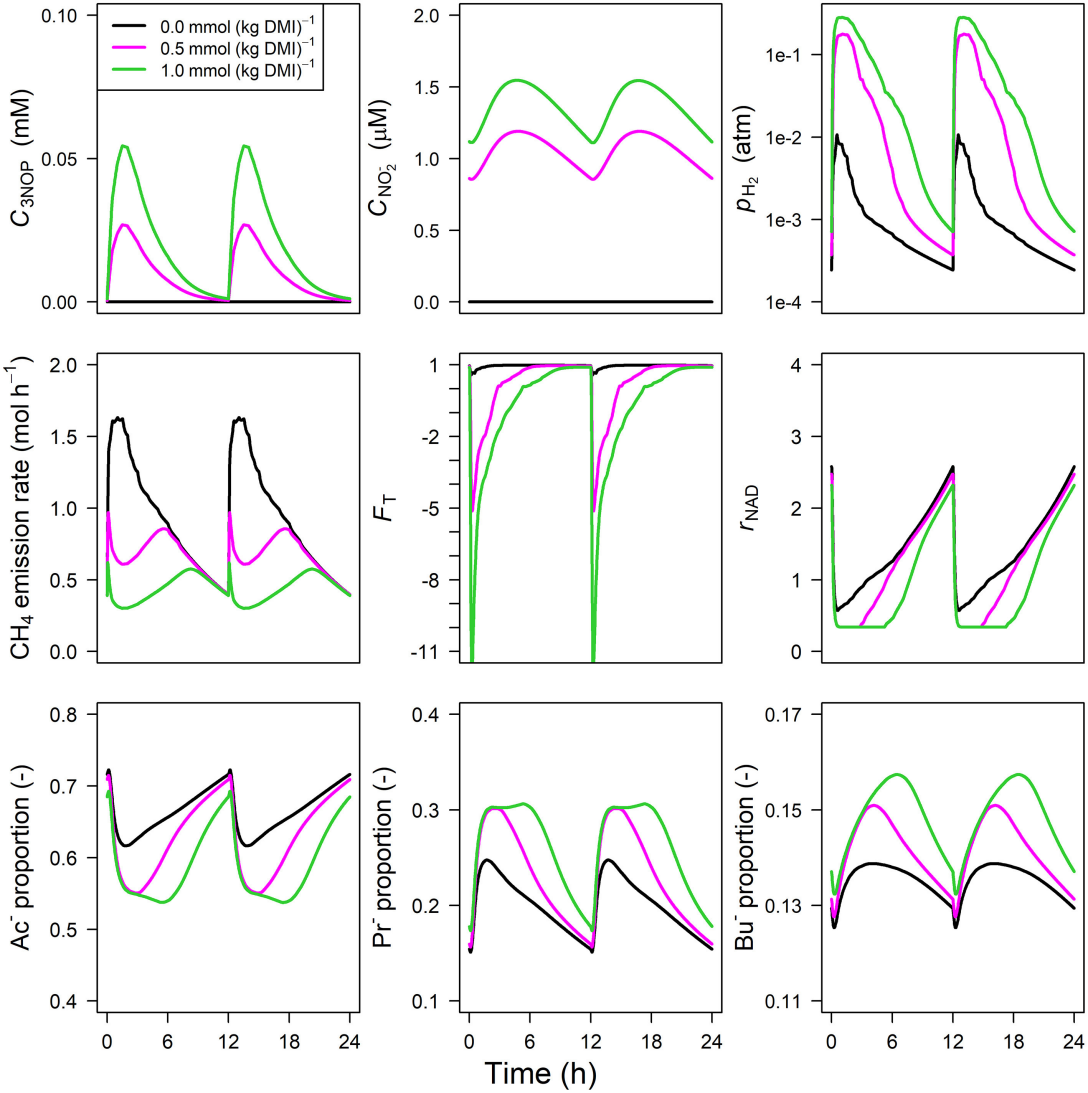
Solutions of the 3-NOP dynamic model with $\text{N}{\text{O}}_{2}^{-}$ representation for 3-NOP concentration [mM], $\text{N}{\text{O}}_{2}^{-}$ concentration [μM], rumen headspace *p*_H_2__ [atm], CH_4_ emission rates [mol·h^−1^], thermodynamic potential factor (*F*_T_; [–]), NAD^+^ to NADH ratio (*r*_NAD_), acetate proportion (Ac^−^), propionate proportion (Pr^−^), and butyrate proportion (Bu^−^).

**Figure 5:**
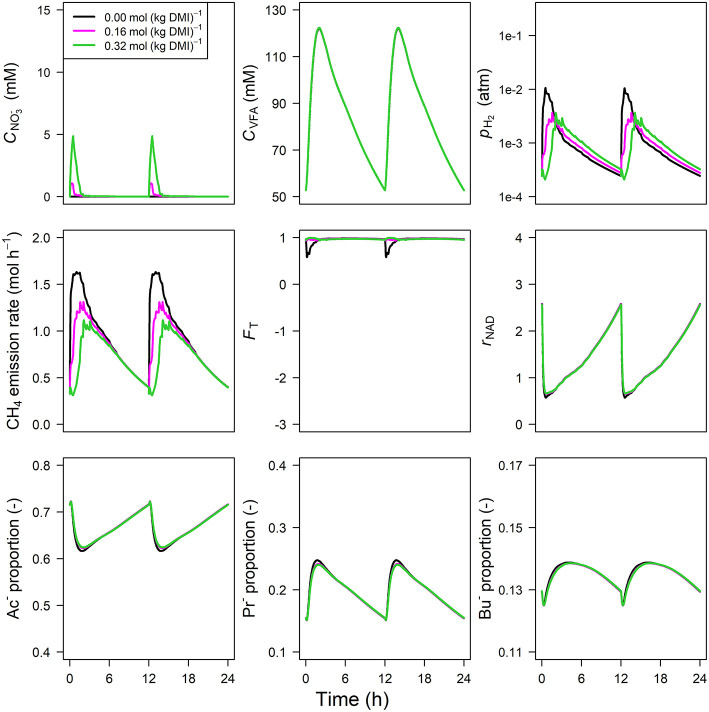
Solutions of the $\text{N}{\text{O}}_{3}^{-}$ dynamic model without $\text{N}{\text{O}}_{2}^{-}$ representation for $\text{N}{\text{O}}_{3}^{-}$ concentration [mM], VFA concentration [mM], rumen headspace *p*_H_2__ [atm], CH_4_ emission rates [mol·h^−1^], thermodynamic potential factor (*F*_T_; [–]), NAD^+^ to NADH ratio (*r*_NAD_), acetate proportion (Ac^−^), propionate proportion (Pr^−^), and butyrate proportion (Bu^−^).

**Figure 6:**
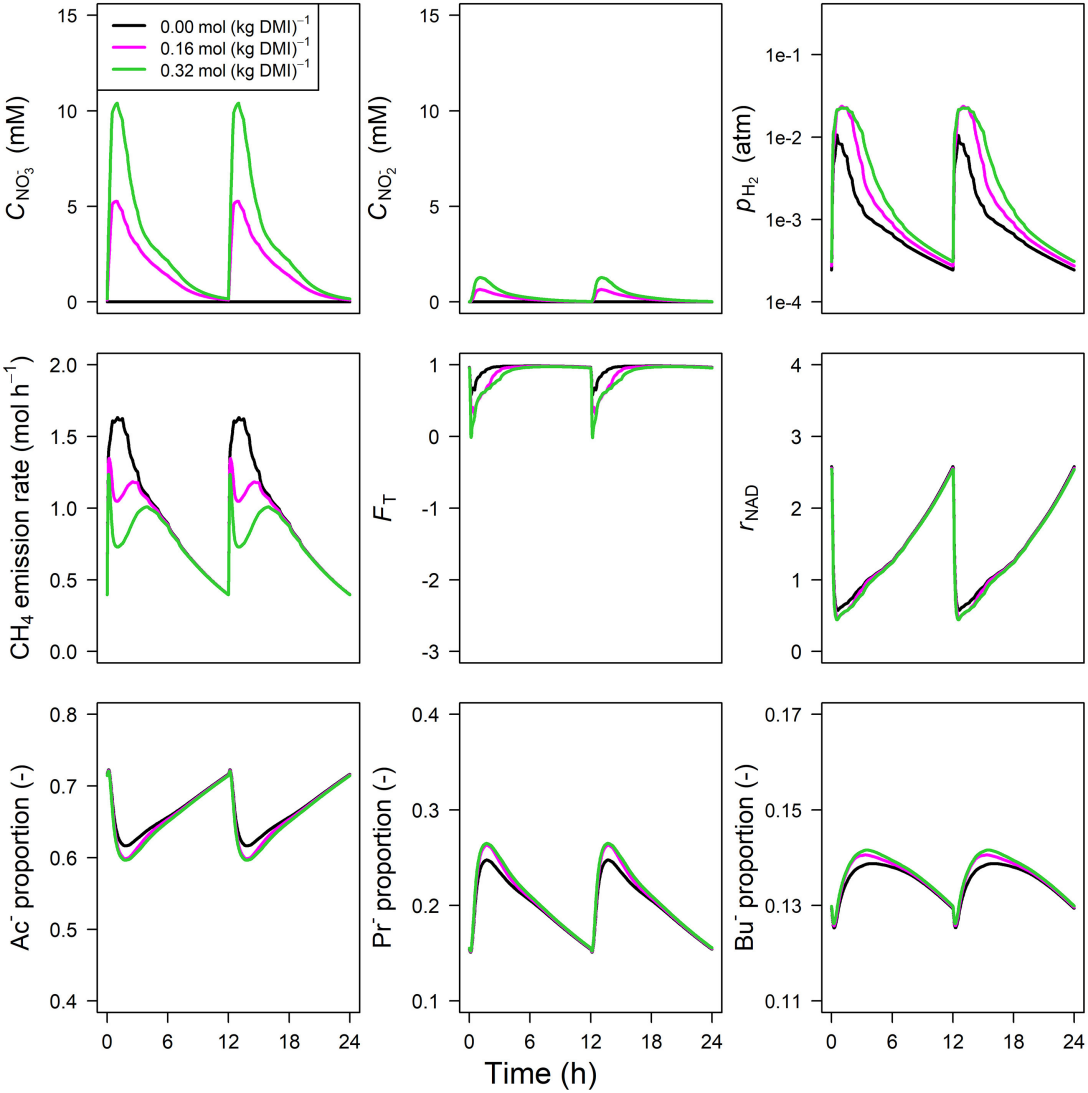
Solutions of the $\text{N}{\text{O}}_{3}^{-}$ dynamic model with $\text{N}{\text{O}}_{2}^{-}$ representation for $\text{N}{\text{O}}_{3}^{-}$ concentration [mM], $\text{N}{\text{O}}_{2}^{-}$ concentration [mM], rumen headspace *p*_H_2__ [atm], CH_4_ emission rates [mol·h^−1^], thermodynamic potential factor (*F*_T_; [–]), NAD^+^ to NADH ratio (*r*_NAD_), acetate proportion (Ac^−^), propionate proportion (Pr^−^), and butyrate proportion (Bu^−^).

Parameter estimates of the optimized parameters of the four different models are provided in Table 3. In response to the assumed feed intake rate and all other parameters that were adopted from Van Lingen et al. (2019), all reference simulations in Figures 3–**6**, i.e., zero inclusion of 3-NOP and $\text{N}{\text{O}}_{3}^{-}$, are identical to the simulations shown in this study by definition. The present 3-NOP model predicts a 3-NOP concentration up to about 0.055 mM at 1.5 h from *in silico* feeding for the highest inclusion rate (Figure 3). Predicted 3-NOP concentrations then steadily approached zero at 12 h at which the next portion of feed was delivered. The diurnal dynamics of the total VFA concentration appeared largely unaffected by the inclusion of 3-NOP, whereas *p*_H_2__ clearly increased in response to 3-NOP inclusion, with a peak of 0.3 atm at about 1 h from feeding for the 1.0 mmol·kg^−1^ inclusion rate. The emission rate of H_2_ followed a similar dynamic pattern as *p*_H_2__ (result not shown). In contrast to the increased peak in *p*_H_2__, the CH_4_ emission rate in response to 3-NOP decreased almost immediately after feeding and then increased to the reference emission rate, while *C*_3NOP_ approached zero. Increased *p*_H_2__ exerted increased thermodynamic inhibition of NADH oxidation, as indicated by the decreased minima of the thermodynamic potential factor (*F*_T_; a dimensionless factor that corrects a predicted kinetic reaction rate for the thermodynamic control exerted; *F*_T_ = 1 indicates no thermodynamic inhibition; *F*_T_ = 0 indicates equilibrium between forward and reverse reaction or, in other words, complete inhibition of the chemical reaction) and the prolonged decrease of *r*_NAD_. It should be noted that for both non-zero inclusion rates of 3-NOP, *r*_NAD_ starts reconditioning toward basal level at about 3 and 5 h from feeding when *F*_T_ is equal to zero (*F*_T_ = 0 indicates neither the forward nor the reverse reaction of NADH oxidation are thermodynamically feasible). The decrease in *r*_NAD_ after feeding was prolonged by 3-NOP supplementation that also resulted in decreased acetate, increase propionate and increased butyrate proportions that were prolonged. Extending the 3-NOP model to the 3-NOP+nitrite model had negligible effect on the dynamics of total VFA concentration (result not shown), whereas non-zero basal $C_{\text{N}{\text{O}}_{2}^{-}}$ and peaks of 0.2 and 0.5 μM at 3.25 and 2.75 h from feeding appeared for the two inclusion rates, respectively (Figure 4). Other dynamics predicted by the 3-NOP+nitrite model appeared similar to the 3-NOP model.

The concentration of $\text{N}{\text{O}}_{3}^{-}$ predicted by the $\text{N}{\text{O}}_{3}^{-}$ model showed an increase from 0 to 1.75 and 5.75 mM in 0.5 h for $\text{N}{\text{O}}_{3}^{-}$ inclusion rates of 0.16 and 0.32 mol·kg^−1^ DMI, respectively, and then steadily approached zero at 12 h at which the next portion of feed was delivered (Figure 5). Peak *p*_H_2__ was clearly decreased and delayed in response to $\text{N}{\text{O}}_{3}^{-}$ inclusion with a *p*_H_2__ value of 2.4×10^−3^ atm at 3.1 h from feeding for the 0.32 mol·kg^−1^ inclusion rate vs. 1 × 10^−2^ atm at 0.5 h for zero $\text{N}{\text{O}}_{3}^{-}$ inclusion. A qualitatively similar decrease was simulated for the emission rate of H_2_ (result not shown). In line with this decrease in H_2_, the CH_4_ emission rate was decreased compared to the reference simulation as well. Decreased *p*_H_2__ alleviated the thermodynamic inhibition of NADH oxidation, as indicated by *F*_T_ approaching one throughout almost the entire 24 h simulation period for the highest $\text{N}{\text{O}}_{3}^{-}$ inclusion rate. The *r*_NAD_ and the proportions of acetate, propionate and butyrate were negligibly affected by the inclusion of $\text{N}{\text{O}}_{3}^{-}$, as was the total VFA concentration. Extending the nitrate model to the nitrate+nitrite model negligibly affected the dynamics of total VFA concentration (result not shown), whereas the $C_{\text{N}{\text{O}}_{2}^{-}}$ diurnal pattern qualitatively followed the $C_{\text{N}{\text{O}}_{3}^{-}}$ diurnal pattern (Figure 6). In contrast to the nitrate model, the nitrate+nitrite model predicted an increase in *p*_H_2__ with a peak of ~2.5×10^−2^ atm from 1 to 2 h from feeding in response to $\text{N}{\text{O}}_{3}^{-}$ inclusion in the diet, whereas a relatively similar decrease in CH_4_ emission rate was simulated. In line with the increase in *p*_H_2__, *r*_NAD_ and the proportions of acetate, propionate and butyrate decreased, decreased, increased and increased, respectively. When zooming in on the highest inclusion rate of $\text{N}{\text{O}}_{3}^{-}$ using the nitrate+nitrite model, 2% passes out from the rumen after reduction to $\text{N}{\text{O}}_{2}^{-}$, 3% is absorbed after reduction to $\text{N}{\text{O}}_{2}^{-}$, 13% passes out from the rumen to the lower gastrointestinal tract, 32% is absorbed, and 51% undergoes complete reduction to NH_3_. These percentages indicate that 51% + 0.25 × (3%+2%) = 52% of the potential of $\text{N}{\text{O}}_{3}^{-}$ as a H_2_ sink is utilized, where 0.25 relates to one of the four H_2_ equivalents for complete reduction of $\text{N}{\text{O}}_{3}^{-}$ are consumed by fermentative microbes. Lastly, a qualitative overview of the output of the four different models in response to 3-NOP and $\text{N}{\text{O}}_{3}^{-}$ supplementation is provided in Table 1B.

### 3.2. Global Sensitivity Analysis

**Figure 7:**
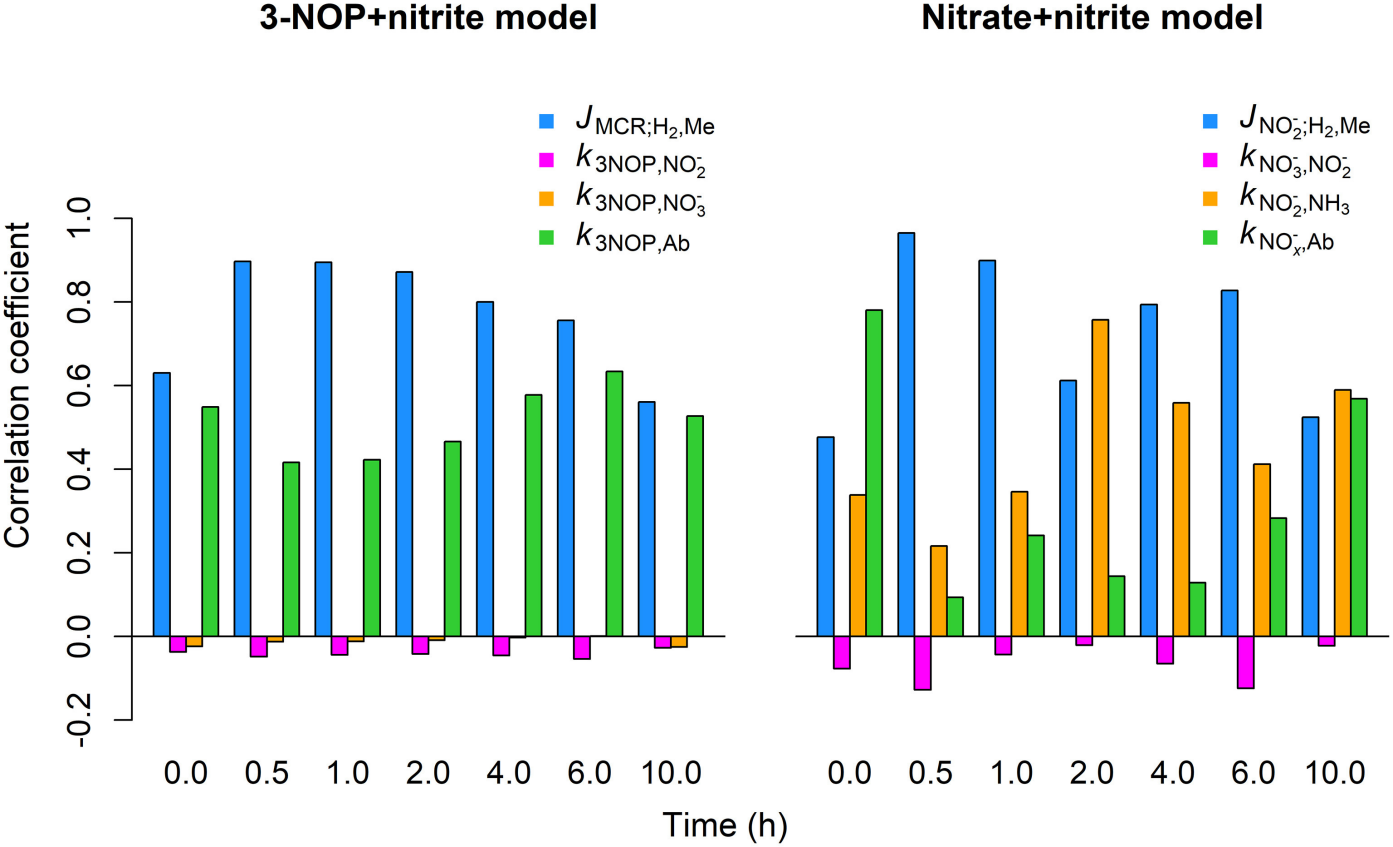
Correlation between CH_4_ emission rate and parameter values obtained from global sensitivity analysis for the 3-NOP+nitrite and nitrate+nitrite model when using inclusion rates of 1.0 mmol·kg^−1^ for 3-NOP and 0.32 mol·kg^−1^ for nitrate and the Van Lingen et al. (2017) feed input. Parameters values were drawn from the interval of 0.75 to 1.25 times their optimum value (see Table 3) using latin hypercube sampling and a sample size of 1,000. Correlation coefficents were calculated at 0, 0.5, 1, 2, 4, 6, and 10 h from the last meal of a 240 h simulation.

The *J*_MCR;_H__2_, Me_ inhibition parameter showed the strongest positive correlation with the CH_4_ emission rate (*r*= 0.6 to 0.90) by the 3-NOP+nitrite model for the different time points for which the global sensitivity analysis was performed (Figure 7). The *k*_3NOP,Ab_ parameter related to 3-NOP reduction also showed positive correlations, although the magnitude of the correlations was slightly stronger for the *J*_MCR;_H__2_, Me_ parameter. The $k_{3\text{NOP},\text{N}{\text{O}}_{2}^{-}}$ absorption parameter was negligibly correlated to CH_4_ emission rate at any of the time points. Correlations between the $k_{3\text{NOP},\text{N}{\text{O}}_{3}^{-}}$ parameter and CH_4_ emission rate were also very minor, |*r*| ≤ 0.10, but were consistently negative. For the nitrate+nitrite model, the $J_{\text{N}{\text{O}}_{2}^{-};{\text{H}}_{2},\text{Me}}$ inhibition parameter showed correlations of 0.61 to 0.97 from 0.5 to 6 h and correlations of approximately 0.5 at 0.0 and 10 h, whereas the $k_{\text{N}{\text{O}}_{2}^{-},\text{N}{\text{H}}_{3}}$ parameter related to $\text{N}{\text{O}}_{2}^{-}$ reduction showed the correlations from roughly 0.22 to 0.76 at the various time points. The $k_{\text{N}{\text{O}}_{3}^{-},\text{N}{\text{O}}_{2}^{-}}$ parameter related to $\text{N}{\text{O}}_{3}^{-}$ reduction showed very weakly negative correlations varying from −0.02 to −0.13. The $k_{\text{N}{\text{O}}_{\text{x}}^{-},\text{Ab}}$ parameter related to absorption of $\text{N}{\text{O}}_{3}^{-}$ and $\text{N}{\text{O}}_{2}^{-}$ had the highest correlations of 0.78 and 0.57 at basal level, that is at 0 and 10 h, respectively, with the correlations at the other times points varying from 0.09 to 0.28.

## 4. Discussion

The present paper presents models for simulating the dynamics of rumen metabolic physiology after supplementing two effective inhibitors of enteric CH_4_ emissions from cattle, viz. 3-NOP and $\text{N}{\text{O}}_{3}^{-}$. It should be noted that 3-NOP is also economically profitable at farm level, whereas this could not be clearly indicated for $\text{N}{\text{O}}_{3}^{-}$ (Alvarez-Hess et al., 2019). Furthermore, $\text{N}{\text{O}}_{3}^{-}$ supplementation may increase the concentration of the $\text{N}{\text{O}}_{2}^{-}$ intermediate to levels that are poisonous to the animal. To the authors' knowledge, the present study is the first effort that describes the metabolism of methanogenic inhibition in the rumen using dynamic mechanistic modeling. Presenting 3-NOP and $\text{N}{\text{O}}_{3}^{-}$ models aids to distinguish the mode of action of decreased CH_4_ caused by supplementation of 3-NOP and $\text{N}{\text{O}}_{3}^{-}$ to diets of cattle and other domestic ruminants, and explores further metabolic implications of H_2_ accumulation and its impact on VFA dynamics. The latter metabolic changes were most clearly indicated by the two 3-NOP models. The 3-NOP to $\text{N}{\text{O}}_{2}^{-}$ conversion rate of the 3-NOP+nitrite model did not affect the inhibition potential of administered 3-NOP, whereas the 3-NOP to $\text{N}{\text{O}}_{3}^{-}$ conversion rate appeared to alleviate methanogenic inhibition. The different metabolic dynamics of the two $\text{N}{\text{O}}_{3}^{-}$ models point to the significance of the impact of $\text{N}{\text{O}}_{2}^{-}$ as an inhibitor of methanogenic archaea, in addition to the metabolic steps that reduce $\text{N}{\text{O}}_{3}^{-}$ to NH_3_ and serve as H_2_ sinks. The present modeling framework by which methanogenesis is inhibited by the concentration of an inhibitor (3-NOP models and nitrate+nitrite model) is possibly applicable to a wider variety of methanogenic inhibitors that are fed to various ruminant species.

### 4.1. Parameter Estimation Procedure

Data availability is an important determinant of model parameter identifiability (e.g., Brun et al., 2001). Data used for parameter estimation of the present models comprised average daily CH_4_ emissions for both 3-NOP models and the nitrate model, whereas data describing diurnal dynamics of H_2_ and CH_4_ emission rates were used for the nitrate+nitrite model. It would be ideal, however, to obtain data that describes the diurnal dynamics of metabolites and also includes rumen 3-NOP, $\text{N}{\text{O}}_{3}^{-}$ and $\text{N}{\text{O}}_{2}^{-}$ concentrations. A dataset that comprises the concentrations of all these metabolites would increase the identifiability of the parameters, particularly of the nitrate+nitrite and 3-NOP+nitrite models for which the $k_{3\text{NOP},\text{N}{\text{O}}_{3}^{-}}$, $k_{3\text{NOP},\text{N}{\text{O}}_{2}^{-}}$, $k_{\text{N}{\text{O}}_{3}^{-},\text{N}{\text{O}}_{2}^{-}}$ and $k_{\text{N}{\text{O}}_{\text{x}}^{-},\text{Ab}}$ parameters were not estimated to data. Such data would likely also increase the accuracy of the simulated diurnal profiles of the various metabolites. Despite a relatively large variation of ruminal $\text{N}{\text{O}}_{3}^{-}$ and $\text{N}{\text{O}}_{2}^{-}$ concentrations across published studies (e.g., Veneman et al., 2015; Wang et al., 2018), $\text{N}{\text{O}}_{3}^{-}$ and $\text{N}{\text{O}}_{2}^{-}$ concentrations of the same study were within the same order of magnitude for both studies. The $\text{N}{\text{O}}_{3}^{-}$ and $\text{N}{\text{O}}_{2}^{-}$ concentrations simulated using the nitrate+nitrite model are in the same order of magnitude as well, suggesting that our $k_{\text{N}{\text{O}}_{3}^{-},\text{N}{\text{O}}_{2}^{-}}$ estimate has a fair degree of accuracy, given that the $k_{\text{N}{\text{O}}_{2}^{-},\text{N}{\text{H}}_{3}}$ was highly identifiable to the diurnal profiles of H_2_ and CH_4_ emission. Although various parameters may not have the utmost accuracy, different estimates may not result in different conclusions being drawn regarding the mechanisms by which CH_4_ production is inhibited and the sensitivity of the CH_4_ emission rate to these parameters may not change and be more related to the overall developed model structures.

### 4.2. Inhibited Methanogenesis and Metabolism

The 3-NOP models predicted increased and decreased emission rates of H_2_ and CH_4_ upon 3-NOP supplementation, respectively, which indicated the model behavior was in line with various responses observed *in vivo* (e.g., Van Gastelen et al., 2020). The present models that are extensions of the Van Lingen et al. (2019) model, which accounts for the thermodynamic control of rumen fermentation by representing a H_2_ pool and the inclusion of NAD^+^ and NADH, predict thermodynamic inhibition of NADH oxidation and next more pronounced minima and maxima in VFA proportions after feeding 3-NOP supplemented feed. These predictions align with changes in VFA proportions that were observed *in vivo* (e.g., Haisan et al., 2014, 2017; Romero-Perez et al., 2015; Lopes et al., 2016). The similar responses of the 3-NOP and 3-NOP+nitrite models and the weakly negative correlation between the CH_4_ emission rate and the $k_{3\text{NOP},\text{N}{\text{O}}_{2}^{-}}$ parameter obtained from the global sensitivity analysis indicate that the rate of $\text{N}{\text{O}}_{2}^{-}$ production from 3-NOP has a minor effect on the inhibition of methanogenesis.

Extension of the nitrate model with a $\text{N}{\text{O}}_{2}^{-}$ representation reversed the pattern of *p*_H_2__ and the H_2_ emission rate in response to $\text{N}{\text{O}}_{3}^{-}$ supplementation. The increased H_2_ emission rate simulated using the nitrate+nitrite model reproduces the *in vivo* experiments used for model calibration (Van Zijderveld et al., 2011; Veneman et al., 2015; Olijhoek et al., 2016), and is also in line with increased dissolved H_2_ concentration observed in faunated and defaunated *in vitro* systems (Wenner et al., 2020). This increase in dissolved concentration and emission rate of H_2_ supports the role of $\text{N}{\text{O}}_{2}^{-}$ as an inhibitor of methanogenesis (Iwamoto et al., 2002), which makes H_2_ accumulate. The positive correlations observed from the global sensitivity analysis for the CH_4_ emission rate with the $J_{\text{N}{\text{O}}_{2}^{-};{\text{H}}_{2},\text{Me}}$ and $k_{\text{N}{\text{O}}_{2}^{-},\text{N}{\text{H}}_{3}}$ parameters point to the significance of the contribution of $\text{N}{\text{O}}_{2}^{-}$ to the inhibition in CH_4_ emission observed upon $\text{N}{\text{O}}_{3}^{-}$ supplementation. The positive relationship between the $k_{\text{N}{\text{O}}_{2}^{-},\text{N}{\text{H}}_{3}}$ parameter and the CH_4_ emission rate suggests that the major mode of action of decreased CH_4_ production after $\text{N}{\text{O}}_{3}^{-}$ supplementation is caused by $\text{N}{\text{O}}_{2}^{-}$ inhibition rather than H_2_ that is consumed by the reduction of $\text{N}{\text{O}}_{2}^{-}$ to NH_3_. The very weakly negative correlations obtained for $k_{\text{N}{\text{O}}_{3}^{-},\text{N}{\text{O}}_{2}^{-}}$ could be associated with decreased CH_4_ emission by both H_2_ sink reinforcement and $\text{N}{\text{O}}_{2}^{-}$ accumulation resulting in inhibited methanogenesis, although the effect may be negligibly small based on the low absolute correlations. If H_2_ sink mechanisms were the key controller of the CH_4_ emission rate, a negative relationship between the $k_{\text{N}{\text{O}}_{2}^{-},\text{N}{\text{H}}_{3}}$ parameter and the CH_4_ emission rate should have been obtained from the global sensitivity analysis, with increased reduction of $\text{N}{\text{O}}_{3}^{-}$ and $\text{N}{\text{O}}_{2}^{-}$ resulting in less CH_4_. However, possibly in line with the low absolute correlations, Welty et al. (2019) only observed a numerical increase in dissolved H_2_ concentration upon $\text{N}{\text{O}}_{3}^{-}$ supplementation to a continuous culture and no increase in H_2_ production. Therefore, the lack of H_2_ accumulation in this specific study does not point to substantial methanogenic inhibition by $\text{N}{\text{O}}_{2}^{-}$ in continuous cultures. Moreover, another possible explanation for unaffected H_2_ concentration or production aligning with the present modeling study might be that their experimental conditions favored a rapid reduction of $\text{N}{\text{O}}_{2}^{-}$ to NH_3_ that alleviated the methanogenic inhibition by $\text{N}{\text{O}}_{2}^{-}$.

In line with Duin et al. (2016), the present 3-NOP+nitrite model also represents $\text{N}{\text{O}}_{3}^{-}$ formation. Nitrate production from 3-NOP would alleviate the methanogenic inhibition as it does not block MCR, indicating that the proportion in which $\text{N}{\text{O}}_{3}^{-}$ and $\text{N}{\text{O}}_{2}^{-}$ are formed from 3-NOP may determine the persistence of the methanogenic inhibition of 3-NOP supplementation to cattle diets. However, the sensitivity analysis did not indicate the formation rates of $\text{N}{\text{O}}_{3}^{-}$ and $\text{N}{\text{O}}_{2}^{-}$ were substantially influential for the area of the parameters space that was explored. Lack of evidence for the presence of $\text{N}{\text{O}}_{3}^{-}$ and $\text{N}{\text{O}}_{2}^{-}$ reductases in rumen methanogens (Greening et al., 2019) may conceptually support the fact that $\text{N}{\text{O}}_{3}^{-}$ formation alleviates methanogenic inhibition, because $\text{N}{\text{O}}_{3}^{-}$ may then not be reduced to $\text{N}{\text{O}}_{2}^{-}$. However, Duin et al. (2016) observed 0.7 mol of $\text{N}{\text{O}}_{3}^{-}$ and 0.2 mol of $\text{N}{\text{O}}_{2}^{-}$ per mol of MCR when titrating with 3-NOP, which then requires one or more alternative mechanisms for the production of $\text{N}{\text{O}}_{3}^{-}$ and $\text{N}{\text{O}}_{2}^{-}$. 1,3-propanediol also being formed from 3-NOP may suggest the production of NO_2_ that is subsequently converted into $\text{N}{\text{O}}_{3}^{-}$ and $\text{N}{\text{O}}_{2}^{-}$. The latter conversion has been described as a disproportionation reaction, which results in equimolar production of $\text{N}{\text{O}}_{3}^{-}$ and $\text{N}{\text{O}}_{2}^{-}$ (e.g., Park and Lee, 1988; Holleman and Wiberg, 2007). The production of 0.7 and 0.2 mol of $\text{N}{\text{O}}_{3}^{-}$ and $\text{N}{\text{O}}_{2}^{-}$, respectively, may suggest either alternative $\text{N}{\text{O}}_{3}^{-}$ production or $\text{N}{\text{O}}_{2}^{-}$ utilization. If MCR deactivation by 3-NOP results in the formation of $\text{N}{\text{O}}_{2}^{-}$ (Duin et al., 2016), MCR deactivation by $\text{N}{\text{O}}_{2}^{-}$ may then result in the formation of NO (disproportionation also described by Park and Lee, 1988), which could explain why more $\text{N}{\text{O}}_{3}^{-}$ than $\text{N}{\text{O}}_{2}^{-}$ was observed. Furthermore, nitrate esters, which include 3-NOP, may hydrolyze and yield $\text{N}{\text{O}}_{3}^{-}$ and an alkanediol (Baker and Easty, 1950, 1952). Although it is unknown if the latter hydrolysis reaction proceeds inside archaeal cells, it describes the production of $\text{N}{\text{O}}_{3}^{-}$ and 1,3-propanediol from 3-NOP.

Nitrite at the outside or inside of archaeal cells will have consequences for the inhibition of archaeal physiology and methanogenesis. Whether or not transportation of $\text{N}{\text{O}}_{2}^{-}$ across archaeal cell membranes takes place affects our understanding of methanogenic inhibition by $\text{N}{\text{O}}_{2}^{-}$ derived from 3-NOP. Cabello et al. (2004) described some archaea, which are not abundant in the rumen, that possess $\text{N}{\text{O}}_{3}^{-}$ transporters and $\text{N}{\text{O}}_{3}^{-}$ and $\text{N}{\text{O}}_{2}^{-}$ reductases. Therewith, these enzymes were not indicated in rumen methanogens. Furthermore, genes for nitrate and nitrite transporters were searched using the IGM/M online database (https://img.jgi.doe.gov/m/; Chen et al., 2019) using “Methanobrevibacter,” “nitrate,” “nitrite,” and “transporter” did not point to any enzyme that possibly facilitates transportation of $\text{N}{\text{O}}_{2}^{-}$ across the archaeal cell membrane, indicating that $\text{N}{\text{O}}_{2}^{-}$ transportation across archaeal cell membranes is unlikely to occur. Nitrite inside archaeal cells, which is formed from 3-NOP that is transported across the archaeal cell membrane, contributes to blocking MCR and enhances methanogenic inhibition (Duin et al., 2016), although this specific study did not investigate if MCR inhibition is the only way in which $\text{N}{\text{O}}_{2}^{-}$ inhibits CH_4_ production. Besides MCR, membrane-associated enzyme complexes catalyze several metabolic steps of the methanogenic pathway in archaea without cytochromes (Thauer et al., 2008), which are the common methanogens in the rumen. Nitrite at the outside of archaeal cells may inhibit the membrane-associated enzyme complexes or disrupt the electron transport system of the membrane (Yang et al., 2016). In contrast to $\text{N}{\text{O}}_{3}^{-}$ supplementation, 3-NOP supplementation results in substoichiometric ruminal concentrations of $\text{N}{\text{O}}_{2}^{-}$, which may indicate that the actual membrane-associated inhibition of methanogenesis is negligible based on the $J_{\text{N}{\text{O}}_{2}^{-};{\text{H}}_{2},\text{Me}}$ parameter for the $\text{N}{\text{O}}_{2}^{-}$ model that is about two orders of magnitude greater than the *J*_MCR;_H__2_, Me_ parameter for the two 3-NOP models. Furthermore, the value of the latter parameter could be taken as an additional indication for absence of $\text{N}{\text{O}}_{2}^{-}$ transportation across archaeal cell membranes, because the methanogenic metabolism may be completely ceased by blocking of MCR if $\text{N}{\text{O}}_{2}^{-}$ concentrations predicted after $\text{N}{\text{O}}_{3}^{-}$ supplementation to cattle diets occur inside archaea. To the authors' knowledge, ceased methanogenic metabolism has not been observed upon ruminal $\text{N}{\text{O}}_{3}^{-}$ supplementation, which may rule out that $\text{N}{\text{O}}_{2}^{-}$ is transported into archaeal cells.

### 4.3. Hydrogen as a Controller of Fermentation

Inhibited methanogenesis resulted in increased *p*_H_2__ and H_2_ emissions from the rumen, as simulated by both 3-NOP models using different inclusion rates as well as implementing methanogenic inhibition by $\text{N}{\text{O}}_{2}^{-}$ when transitioning from the nitrate to the nitrate+nitrite model. Increased *p*_H_2__ exerted inhibition of NADH oxidation, which resulted in decreased proportions of acetate and increased proportions of propionate and butyrate (Van Lingen et al., 2016, 2019). These respective shifts in VFA proportions in response to *p*_H_2__, which are also described by Janssen (2010), align with *in vivo* observations (Haisan et al., 2014, 2017; Lopes et al., 2016) for 3-NOP, whereas VFA proportions in response to $\text{N}{\text{O}}_{3}^{-}$ supplementation seem less consistent in the literature. Observations were that acetate proportion was unaffected or increased, propionate proportion was unaffected, increased or decreased, and butyrate proportion was unaffected or increased across various studies (e.g., Guyader et al., 2015; Troy et al., 2015; Veneman et al., 2015; Olijhoek et al., 2016; Wang et al., 2018). This somewhat diverse picture in response to $\text{N}{\text{O}}_{3}^{-}$ may be related to the methanogenic inhibition that is likely employed, which is adverse to the H_2_ sink mechanism in relation to thermodynamic inhibition of NADH oxidation and associated VFA proportions. Ruminal conditions that control the favorability of $\text{N}{\text{O}}_{2}^{-}$ reduction may determine the occurrence of the H_2_ sink mechanism and the methanogenic inhibition by $\text{N}{\text{O}}_{2}^{-}$ mechanism. A mixed culture *in vitro* experiment by Anderson et al. (2016) indicated a decreased acetate to propionate ratio and an increased headspace *p*_H_2__ in response to increased $\text{N}{\text{O}}_{3}^{-}$ supplementation, whereas these changes were impaired when the mixed culture was also inoculated with *Denitrobacterium detoxificans*, despite a more pronounced decrease of headspace CH_4_ partial pressure. This inoculation may have stimulated the reduction of $\text{N}{\text{O}}_{2}^{-}$ and alleviated methanogenic inhibition and H_2_ accumulation, and next affected the production of the different VFA. Therefore, these observations will likely be reproduced by a nitrate model such as the present nitrate+nitrite model in which both the H_2_ sink mechanism and the nitrite inhibition of methanogenesis mechanism are implemented.

Thermodynamic inhibition of NADH oxidation was greatest for the highest *p*_H_2__ that was simulated and changed VFA proportions the most, perhaps more than observed *in vivo*. Electron-bifurcating hydrogenases that are able of reoxidizing NADH oxidation (e.g., Buckel and Thauer, 2018), were found to be the primary mediators of H_2_ production by a metatranscriptomics analysis, but this analysis did not indicate that these hydrogenases were expressed differently in high and low CH_4_ emitting sheep (Greening et al., 2019). No differences between hydrogenase enzyme expressions in these two groups of sheep may not suggest that VFA proportions in ovine rumens were changed (Van Lingen et al., 2016) and also that the present modeling framework of rumen fermentation metabolism that did predict changes in VFA proportions is too simple. However, Greening et al. (2019) did not relate actual H_2_ emissions to enzyme expressions, nor were their samples collected from animals that were fed diets known to induce inhibition of methanogenic archaea, which point to the need for future studies that explore these relationships. Nonetheless, the latter recent study did report evidence for differences in enzyme expression associated with various alternative H_2_ utilizing pathways in high and low CH_4_ emitting sheep. Besides decreased expression of methanogenic enzymes, they reported increased expression of enzymes that mediate fumarate reduction. Fumarate reduction produces succinate, which is a precursor of propionate. Therefore, increased fumarate reduction upon elevated *p*_H_2__ is expected to stimulate propionate production in the rumen, which qualitatively supports the present model predictions of increased propionate proportions upon feeding dietary substrate that induces methanogenic inhibition. Furthermore, a decrease in H_2_ recovered as the sum of propionate, butyrate, H_2_ and CH_4_ was observed when inhibiting methanogenesis in both batch and continuous culture (Ungerfeld, 2015), although the specific energetic benefits of methanogenic inhibition depended on the type and concentration of the inhibitor and on the *in vitro* system.

A more exhaustive metabolic framework of ruminal H_2_ dynamics may comprise more than the key mechanism by which hydrogenases produce H_2_ and mediate NADH oxidation. Ungerfeld (2015) speculated that H_2_ was incorporated in formate and microbial biomass, and perhaps taken away via reductive acetogenesis in continuous cultures. For the latter H_2_ utilizing pathway, the *p*_H_2__ threshold may be as high as 2.5×10^−3^ atm (Poehlein et al., 2012). Administration of methanogenic inhibitors to the rumen increases the number of hours per day that this threshold is exceeded and may, therefore, stimulate reductive acetogenesis. Upon supplementating bromochloromethane as an methanogenic inhibitor to goats, a metagenomic analysis indicated that, apart from increased *Prevotella* and *Selenomonas* species that are able to produce propionate using the randomizing pathway, reductive acetogenic populations were also affected significantly suggesting that they provide minor contributions to the redirection of H_2_ (Denman et al., 2015). In the previously cited metatranscriptomics analysis for sheep rumens (Greening et al., 2019), reductive acetogenesis was indicated and enzyme expression was negatively correlated to CH_4_ yield. Therefore, the incorporation of the reductive acetogenic pathway in the present models may shed further light on the metabolic dynamics in the rumen upon supplementation of inhibitors. However, further studies are required to discover other so far unidentified H_2_ sinks for a better understanding of the metabolic pathways involved in H_2_ production and utilization (Guyader et al., 2017).

### 4.4. Summary of Main Findings

In conclusion, both 3-NOP models and the nitrate+nitrite model predicted that the H_2_ emission rate and *p*_H_2__ increased with the inclusion rate of 3-NOP and $\text{N}{\text{O}}_{3}^{-}$, whereas a decreased CH_4_ emission rate was simulated for these supplements. Omission of the $\text{N}{\text{O}}_{2}^{-}$ state variable from the 3-NOP model did not qualitatively change the overall dynamics of H_2_ and CH_4_ emission and other metabolites. However, omitting the $\text{N}{\text{O}}_{2}^{-}$ state variable from the $\text{N}{\text{O}}_{3}^{-}$ model substantially changed the dynamics of H_2_ and CH_4_ emissions indicated by a decrease in the emission rates of these two gases after feeding. Increased *p*_H_2__ induced by methanogenic inhibition, after 3-NOP supplementation particularly, resulted in decreased proportions of acetate and increased proportions of propionate and butyrate, although the incorporation of alternative H_2_ consuming pathways may contribute to less pronounced responses in VFA proportions being predicted. The findings of this modeling study provide deeper insights into the metabolic physiology of ruminal bacteria, protozoa and archaea in response to two effective inhibitors of enteric CH_4_ production. These insights will contribute to a better use of antimethanogenic additives and therefore help reducing enteric CH_4_ production and the total ecological footprint of ruminant livestock production in the future.

## Data Availability Statement

R code and data files that support the model simulations of this study can be found online at the GitHub repository through: https://github.com/linge006/Modeling-inhibited-methanogenesis.

## Author Contributions

HL designed the research, performed all simulations of this study, and wrote the paper. HL, DY-R, and MK did the conceptualization. JF, EK, and MK supervised the work. EK and MK were responsible for the project administration. All authors reviewed drafts of the manuscript and approved the final version.

## Conflict of Interest

MK is affiliated with DSM Nutritional products, which is the funder of the present study and patented 3-NOP. The remaining authors declare that the research was conducted in the absence of any commercial or financial relationships that could be construed as a potential conflict of interest.

## Publisher's Note

All claims expressed in this article are solely those of the authors and do not necessarily represent those of their affiliated organizations, or those of the publisher, the editors and the reviewers. Any product that may be evaluated in this article, or claim that may be made by its manufacturer, is not guaranteed or endorsed by the publisher.
